# Comparative Genomics of Marine Sponge-Derived *Streptomyces* spp. Isolates SM17 and SM18 With Their Closest Terrestrial Relatives Provides Novel Insights Into Environmental Niche Adaptations and Secondary Metabolite Biosynthesis Potential

**DOI:** 10.3389/fmicb.2019.01713

**Published:** 2019-07-26

**Authors:** Eduardo L. Almeida, Andrés Felipe Carrillo Rincón, Stephen A. Jackson, Alan D. W. Dobson

**Affiliations:** ^1^School of Microbiology, University College Cork, Cork, Ireland; ^2^Environmental Research Institute, University College Cork, Cork, Ireland

**Keywords:** comparative genomics, *Streptomyces*, environmental adaptation, marine sponge bacteria, secondary metabolite biosynthetic gene clusters, single molecule real-time sequencing

## Abstract

The emergence of antibiotic resistant microorganisms has led to an increased need for the discovery and development of novel antimicrobial compounds. Frequent rediscovery of the same natural products (NPs) continues to decrease the likelihood of the discovery of new compounds from soil bacteria. Thus, efforts have shifted toward investigating microorganisms and their secondary metabolite biosynthesis potential, from diverse niche environments, such as those isolated from marine sponges. Here we investigated at the genomic level two *Streptomyces* spp. strains, namely SM17 and SM18, isolated from the marine sponge *Haliclona simulans*, with previously reported antimicrobial activity against clinically relevant pathogens; using single molecule real-time (SMRT) sequencing. We performed a series of comparative genomic analyses on SM17 and SM18 with their closest terrestrial relatives, namely *S. albus* J1074 and *S. pratensis* ATCC 33331 respectively; in an effort to provide further insights into potential environmental niche adaptations (ENAs) of marine sponge-associated *Streptomyces*, and on how these adaptations might be linked to their secondary metabolite biosynthesis potential. Prediction of secondary metabolite biosynthetic gene clusters (smBGCs) indicated that, even though the marine isolates are closely related to their terrestrial counterparts at a genomic level; they potentially produce different compounds. SM17 and SM18 displayed a better ability to grow in high salinity medium when compared to their terrestrial counterparts, and further analysis of their genomes indicated that they possess a pool of 29 potential ENA genes that are absent in *S. albus* J1074 and *S. pratensis* ATCC 33331. This ENA gene pool included functional categories of genes that are likely to be related to niche adaptations and which could be grouped based on potential biological functions such as osmotic stress, defense; transcriptional regulation; symbiotic interactions; antimicrobial compound production and resistance; ABC transporters; together with horizontal gene transfer and defense-related features.

## Introduction

With the emergence and rapid spread of antibiotic resistant microorganisms, displaying resistance to many currently available antibiotics, a concerted effort continues to be needed to discover novel antimicrobial agents (Thabit et al., 2015; Rolain et al., 2016). Members of the *Streptomyces* genus are also known to produce a broad range of other natural products (NPs) which possess immunosuppressant, anti-fungal, anti-cancer, anti-parasitic and anti-thrombotic activities (Hwang et al., 2014; Ser et al., 2017). However, the frequent re-discovery of previously characterized bioactive compounds from terrestrial *Streptomyces*, has somewhat limited the interest of researchers in terrestrial ecosystems as potential reservoirs for novel biomolecules (Yagüe et al., 2012; Dalisay et al., 2013; Paulus et al., 2017). Instead, interest has begun to focus on the isolation of *Streptomyces* from other environmental niches; with *Streptomyces* involved in symbiotic relationships or associated with plants, insects, fungi, lichens, sea-cucumbers, seaweeds and marine sponges also attracting increased attention as potential reservoirs for these types of bioactive molecules (Motohashi et al., 2010; Seipke et al., 2012; van der Meij et al., 2017). The ability of these *Streptomyces* to colonize such a wide variety of hosts is due in part to their ability to produce useful NPs, such as antimicrobials which help their hosts defend themselves against predators or pathogenic bacteria and fungi (Adnani et al., 2017; van der Meij et al., 2017).

Marine ecosystems are attracting particular attention, where extreme and rapidly changing environmental conditions such as differences in pressure, salinity, pH, light intensity, temperature and oligotrophic conditions are believed to be linked to secondary metabolites production (Abdelmohsen et al., 2014; van der Meij et al., 2017). In this respect, marine ecosystems have been a particularly fruitful source of *Streptomyces* strains which have the potential to produce new bioactive NPs (Hassan et al., 2017; Jin et al., 2018; Xu et al., 2018), with marine *Streptomyces* being isolated from seashores, coastal waters, bottom sediments, fishes, molluscs, sponges, seaweeds and mangroves (Manivasagan et al., 2014; Ser et al., 2017).

Marine sponges (phylum *Porifera*) in particular are known to be a rich source of bioactive compounds, many of which are produced by the bacteria which reside within the sponge host (Abdelmohsen et al., 2014; Fuerst, 2014). Many of these bioactives have antimicrobial activities, making these sponge-associated microbial and fungal communities a potentially valuable source of novel antimicrobials (Baker et al., 2009; Flemer et al., 2012; Hoppers et al., 2015; Indraningrat et al., 2016; Jackson et al., 2018). While sponge bacteria-derived antimicrobial compounds have to date been identified from 35 different genera, the most predominant producing genera include *Streptomyces*, *Pseudovibrio* and *Bacillus* strains (Indraningrat et al., 2016). Of these, *Streptomyces* are the predominant genus, producing around 30% of the compounds identified to date (Indraningrat et al., 2016). Good examples of bioactive compounds produced from *Streptomyces* associated with marine sponges include: mayamycin, produced by *Streptomyces* sp. HB202 isolated from *Halichondria panicea* (Schneemann et al., 2010); the naphthacene glycoside SF2446A2, produced by *Streptomyces* sp. RV15 isolated from *Dysidea tupha* (Reimer et al., 2015); and Petrocidin A, produced by *Streptomyces* sp. SBT348 isolated from *Petrosia ficiformis* (Cheng et al., 2017).

As previously mentioned, in addition to marine sponges, many *Streptomyces* strains have also evolved symbiotic relationships with plants, fungi, and insects, amongst others; and there is increasing evidence that the host may control which metabolic pathways are activated within their symbionts, such as in the tunicate *Lissoclinum patella* and the squid *Euprymna scolopes* (Kwan et al., 2014; Gromek et al., 2016). In *Streptomyces* spp., it is clear that not only do they benefit from the resources of the hosts they interact with, but that these interactions control the expression of secondary metabolite biosynthetic gene clusters (smBGCs); thereby promoting the high degree of chemical diversity observed in the secondary metabolites being produced by these organisms (van der Meij et al., 2017). An example is the recent report that exposure of the endosymbiont *Streptomyces* ACT-52A to *Aplysilla rosea* promoted production of bioactive compounds with antibacterial activity (Mehbub et al., 2016). The factors involved in controlling the expression of these smBGCs are likely to be quite diverse, given the large degree of variability in the habitats and potential hosts, and how they are presumably influencing the secondary metabolite biosynthetic potential of *Streptomyces* symbionts (Adnani et al., 2017). Thus, it is clear that an increased knowledge of the genetics underpinning the interactions and signaling between the sponge host and the symbiont is required, through identification of smBGCs in the genomes of these sponge associated *Streptomyces* strains, coupled with identification of potential environmental “triggers” from the sponge, from other sponge endosymbionts, and/or from the surrounding marine environment that may regulate transcription of these smBGCs (Mehbub et al., 2016; Adnani et al., 2017; van der Meij et al., 2017).

To this end, we recently sequenced the genomes of 13 *Streptomyces* spp. isolated from both shallow water and deep-sea sponges, that displayed antimicrobial activities against a number of clinically relevant bacterial and yeast species (Kennedy et al., 2009; Jackson et al., 2018). Using the antiSMASH (antibiotics and Secondary Metabolite Analysis Shell) software (Blin et al., 2017), the strains were found to host abundant smBGCs which potentially encode polyketides, non-ribosomal peptide synthases (NRPS), siderophores, lantipeptides, and bacteriocins (Jackson et al., 2018). Thus, these strains appear to be a promising source of novel bioactive secondary metabolites, as the abundance and diversity of smBGCs displayed high degrees of novelty. In addition, the strains were enriched for genes potentially involved in the biosynthesis and transport of compatible solutes and for heat-shock proteins, genes which are typically associated with marine adaptations (Penn and Jensen, 2012; Tian et al., 2016).

Around sixty marine adaptation genes (MAGs) have previously been proposed for the obligate marine actinomycete genus *Salinispora*, with the function of these genes being associated with electron transport, sodium and ABC transporters, together with channels and pores (Penn and Jensen, 2012). Even though sponge-associated *Streptomyces* are marine bacteria, the environmental niche occupied by these organisms differs quite markedly from *Salinispora*, thus the genetic adaptions may not necessarily be similar. This was confirmed by the Zotchev group, when the draft genome of two sponge associated *Streptomyces* strains where analyzed for MAGs, revealing the presence of only seven of the *Salinispora* MAG gene pool (Ian et al., 2014). They suggested that specific marine sponge genetic adaptations may exist, given that different genes were identified in these sponge-associated *Streptomyces* which were absent in their soil counterparts (Ian et al., 2014). However, drawing conclusions for these genetic adaptations is quite difficult due to the limited number of sponge-associated *Streptomyces* genomes that are currently available. To this end, we sequenced the genomes of *Streptomyces* strains SM17 and SM18, two of the aforementioned 13 sponge-derived *Streptomyces* spp. that had displayed antimicrobial activity, using the PacBio RSII Single Molecule, Real-Time (SMRT) sequencing platform. This allowed us to study not only the smBGCs that these bacteria possess, but also other genetic characteristics that may be involved in their life cycle; such as for example adaptation to the marine environment and symbiosis. By employing comparative genomics, we compared the genomes of these strains with their most closely related terrestrial type-strain relatives, with complete genomes available in the GenBank database (namely *S. albus* J1074 for SM17 and *S. pratensis* ATCC 33331 for SM18), in an attempt to identify genes potentially associated with ENA, together with genes encoding potentially novel smBGCs.

## Materials and Methods

### Bacterial Strains, Maintenance and Differential Growth Assessment

The SM17 and SM18 strains were isolated from the marine sponge *Haliclona simulans* (Kilkieran Bay, Galway, Ireland), as previously described (Kennedy et al., 2009). The *S. albus* J1074 strain was provided by Dr. Andriy Luzhetskyy (Helmholtz Institute for Pharmaceutical Research Saarland, Germany), while *S. flavogriseus*/*S. pratensis* ATCC 33331 was obtained from the American Type Culture Collection (ATCC Inc., United States). SM17, SM18, *S. albus* J1074 and *S. flavogriseus*/*S. pratensis* ATCC 33331 spores were propagated on mannitol-soya (MS) agar medium at 28°C for 8–10 days and stored in 20% glycerol at −80°C. Strains were cultivated on ISP2 and ISP2 plus artificial sea water (ASW) medium when indicated, for differential growth analysis. The ASW was obtained by adding 3% Instant Ocean^®^ Sea Salt (Instant Ocean Inc., United States) to the medium. It is important to note that the ATCC 33331 strain, due to a more recent taxonomy classification (Rong et al., 2013), is described with two different names: in GenBank as *S. pratensis* ATCC 33331 (new classification), and in the ATCC^®^ culture collection as *S. flavogriseus* ATCC 33331 (old classification). From now on, the ATCC 33331 isolate will be referred to as *S. pratensis* ATCC 33331.

### Genome Sequencing, Assembly and Annotation

Biomass from the SM17 and SM18 strains was obtained after cultivation on TSB medium for 3 days at 28°C and 220 rpm. Genomic DNA from SM17 was isolated using the DNeasy Blood & Cell Culture DNA Midi Kit (Qiagen Inc.); and by using the phenol-chloroform-isoamyl alcohol extraction method for SM18 (Wilson, 2001). Genome sequencing was performed by Macrogen (Seoul, South Korea), using the PacBio RSII sequencing platform.

The PacBio raw reads were processed and quality filtered using the BamTools toolkit v2.4.1 (subread length >1000, subread quality >0.75) (Barnett et al., 2011). The genome assemblies were performed using the Canu v1.7 software (Koren et al., 2017), followed by assembly polishing using Quiver v2.1.0 (Pacific Biosciences Inc). The assembly coverage check was performed using the BBMap program v37.90^1^. Genome assembly statistics were calculated using the QUAST v4.6.3 program (Gurevich et al., 2013). Genome annotation was performed using the Prokka v1.12 program for this study’s analyses (Seemann, 2014), and with the NCBI Prokaryotic Genome Annotation Pipeline for data submission on the GenBank database (Tatusova et al., 2016; Benson et al., 2018). Prediction of smBGCs was performed using the antiSMASH 4 software (Blin et al., 2017). Similarity clustering of smBGCs families was performed using the Biosynthetic Genes Similarity Clustering and Prospecting Engine (BiG-SCAPE, version 2018100) (Navarro-Muñoz et al., 2018) and Cytoscape (v3.7.1) (Shannon et al., 2003), with annotations based on the Minimum Information about a Biosynthetic Gene cluster (MIBiG) repository (v1.4) (Medema et al., 2015). Genome maps were generated using the Artemis v17.0.1 and the DNAPlotter v17.0.1 programs (Rutherford et al., 2000; Carver et al., 2009). Proteins of interest were manually annotated using the NCBI BLAST tool; the GenBank database; and the Conserved Domain Database (CDD) (Johnson et al., 2008; Marchler-Bauer et al., 2015; Benson et al., 2018).

### Comparative Genomics

The closest reference strains for the sponge-derived isolates SM17 and SM18 were determined by employing a phylogenetic analysis performed in two steps: (1) based on the 16S rRNA sequence of the SM17 and SM18 isolates, we picked the top 30 most similar *Streptomyces* species to each of the isolates (for a total of 60 genomes from the database), with complete genome available in GenBank (Benson et al., 2018), using the NCBI BLAST tool (Johnson et al., 2008) (2) we then performed a phylogenetic analysis employing concatenated sequences (Gadagkar et al., 2005) of the 16S rRNA and the housekeeping genes *atpD* (ATP synthase subunit beta), *gyrB* (DNA gyrase subunit B), *recA* (recombinase RecA), *rpoB* (DNA-directed RNA polymerase subunit beta), and *trpB* (tryptophan synthase beta chain), of the SM17 and SM18 strains, plus the previously determined top 60 most similar *Streptomyces* species. Alignment of the concatenated sequences was performed using the MAFFT program (Katoh and Standley, 2013), and phylogeny was determined using the MrBayes program (Ronquist et al., 2012), applying the General Time Reversible (GTR) model of nucleotide substitution with gamma-distributed rates across sites with a proportion of invariable sites (Waddell and Steel, 1997), and an average standard deviation of split frequencies cut off of 0.01. The final condensed tree, with a posterior probability cut off of 95%, was generated using MEGA X (Kumar et al., 2018) and Inkscape^2^. To further support genomic similarities between the SM17 and SM18 strain and their closest type-strain terrestrial relative determined with the phylogeny analysis, alignments of the individual housekeeping genes were performed and sequence similarity was determined, using the NCBI BLAST tool (Johnson et al., 2008); and whole genome nucleotide alignments were performed using the MUMmer 3.0 program (Kurtz et al., 2004). Plasmids sequences were determined by similarity searches in the GenBank database (Benson et al., 2018). Orthologous gene analysis was performed using the Roary v3.12.0 program, with an identity cut-off set to 50% (Page et al., 2015). The Roary outputs were processed using the R software environment in the RStudio IDE (Racine, 2012; RStudio Team, 2015; R Core Team, 2018), with data frame handling using the plyr package (Wickham, 2011); and Venn diagrams generated using the venn package (Dusa, 2018).

### Accession Numbers

The complete genome sequences of SM17, SM18, and the SM17 plasmid sequences pSM17A, pSM17B, pSM17C, have been deposited in GenBank under the accession numbers CP029338, CP029342, CP029339, CP029340, and CP029341, respectively. The closest reference genomes used in this study for comparative purposes were *S. albus* J1074 (accession no. CP004370.1) and *S. pratensis* ATCC 33331 (accession no. CP002475.1).

## Results and Discussion

### Genome Sequencing and Assembly

The genomes of the marine sponge-derived *Streptomyces* spp. isolates SM17 and SM18 were sequenced using the PacBio RSII sequencing platform, which generated a total of 140,538 and 87,756 subreads respectively, after adapter removal and quality/length filtering (Table 1A). The PacBio sequencing provided long read lengths, averaging 9,702 and 8,923 bp for SM17 and SM18, respectively. Combining the large number of reads and their long length, an approximate sequencing coverage of 194× and 101× was obtained for SM17 and SM18, respectively.

**TABLE 1A:** General characteristics of the SM17 and the SM18 genomes.

	**SM17**	**SM18**
Genome size (bp)	7,179,914^*^	7,703,166
Number of subreads	140,538	87,756
Average subread length (bp)	9,702	8,923
Approximate average sequencing coverage (fold)	194	101
GC content (%)	73.35	71.84
Number of contigs	4^∗∗^	1
N50	6,975,788	7,703,166
L50	1	1
Number of coding sequences	6,181	6,670
Number of rRNAs	21	18
Number of tRNAs	78	82
Number of tmRNAs	1	1

For SM17, the statistics include the sequence of the chromosome in addition to the sequences determined to represent three plasmids, hence the ^*^ >7 Mb genome size and the ^∗∗^total of 4 contigs. No plasmids were identified in the SM18 strain, so the statistics represented above are for the chromosome sequence. No gaps or ambiguous bases (Ns) are present in the final genome assemblies.

The genome assemblies for both isolates were of a very high quality, resulting in single contig assemblies of the chromosomes, without gaps or ambiguous bases (Ns), with a total genome size comprising of 7,179,914 bp (including plasmids sequences, with 6,975,788 bp for the chromosome alone) for SM17; and 7,703,166 bp (without plasmids) for SM18 (Table 1A). High quality genome assemblies are highly advantageous for determining the core genome; identifying genome sequence and structure variants; analyzing gene acquisition and duplication; together with exploring the potential presence of smBGCs at a genetic level, which is particularly relevant for studies on the *Streptomyces* genus (Bentley et al., 2002; Schmid et al., 2018). Although a few marine *Streptomyces* spp. isolates have recently had their genomes sequenced, the majority of these consist of considerably fragmented sequences due to the complexity of the genome assemblies; which to a large extent hinders an in-depth analysis of these organisms at a genomic level, particularly with respect to analyzing the presence of smBGCs (Gomez-Escribano et al., 2015; Jackson et al., 2018). To our knowledge, this is one of the first studies to report the complete genome sequence of marine sponge-derived *Streptomyces* spp. isolates.

The sequencing approach employed allowed the identification of plasmids in the SM17 isolate – pSM17A, pSM17B, and pSM17C (Table 1B). A series of factors led to their classification as plasmids, instead of simply fragments of the chromosome. Firstly, the contigs were much smaller than the super contig determined to be the chromosome: 153,923 bp, 28,056 bp, and 22,147 bp, respectively, when compared to 6,975,788 bp for the chromosome. In addition, their GC content varied from that of the chromosome, which is characteristic of exogenous and plasmid DNA (Nishida, 2012). The approximate sequencing coverage of the sequences was also varied, which is an indicator of differences in the copy number of the plasmid molecules, with pSM17B having a considerably larger coverage of 548×, as opposed to 170× for pSM17A and 95× for pSM17C (Rasko et al., 2007). Finally, they were determined to share high sequence identity to other plasmids from *Streptomyces* spp. deposited in the GenBank database, as shown in Table 1B (Guo et al., 2011; Wang et al., 2012; Liu et al., 2016).

**TABLE 1B:** General characteristics of the SM17 chromosome and the three linear plasmids detected in the genome assembly.

	**SM17 chromosome**	**Plasmid pSM17A**	**Plasmid pSM17B**	**Plasmid pSM17C**
Size (bp)	6,975,788	153,923	28,056	22,147
Approximate coverage (fold)	148	170	548	95
GC content (%)	73.43	69.9	72.68	74.33
Number of coding sequences (hypothetical proteins)	5,972 (2,465)	170 (145)	30 (24)	24 (21)
Top BLASTN hit	–	*Streptomyces* sp. HK1 plasmid pSHK1 (accession no. EU372836.1)	*Streptomyces* sp. Y27 plasmid pWTY27 (accession no. GU226194.2)	*Streptomyces* sp. FR-008 plasmid pSSFR2 (accession no. CP009804.1)

**FIGURE 1:**
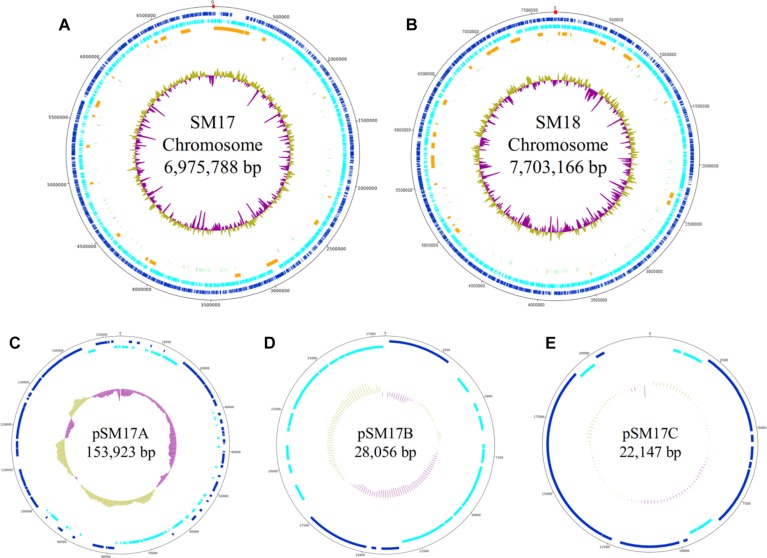
Genome maps of the SM17 and the SM18 chromosomes **(A,B)**, and the SM17 plasmids pSM17A **(C)**, pSM17B **(D)**, pSM17C **(E)**, generated using the Artemis and DNAplotter programs. All the molecules were *in silico*-determined to be linear, although they are represented in a circular fashion, and the sizes are not representative of the scale. The following are represented from the outer to the inner circles: the nucleotide position; coding sequences (CDSs) in the forward strand (in blue); CDSs in the reverse strand (in cyan); regions of putative secondary metabolite biosynthetic gene clusters (smBGCs, in orange); tRNA and rRNA genes (in gray and green, respectively); GC% plot on default settings (above average in olive and below average in purple). In **(A,B)**, detailed in red are the regions determined to be the terminal inverted repeats (TIRs).

Potential Terminal Inverted Repeats (TIRs) with an estimated size of approximately 13.4 kb and 14.6 kb were identified in both the SM17 and SM18 chromosomes respectively, using a reciprocal BLASTN approach at the ends of the chromosome sequences (Gomez-Escribano et al., 2015). The *Streptomyces* genus is known to possess linear chromosomes with TIRs, with lengths varying among species; ranging from 14 bp in *Streptomyces hygroscopicus* 5008 to over 1 Mbp in *S. coelicolor* (Weaver et al., 2004; Wu et al., 2012). Although TIRs are commonly encountered in *Streptomyces* spp., their function has not yet been definitively proven, with suggested roles been proposed including chromosome stability, replication and recombination; and genome plasticity (Volff et al., 1997; Goshi et al., 2002; Choulet et al., 2006a,b; Lin et al., 2009). The main genomic features of SM17 and the three plasmids, and SM18 (number of base pairs, coding sequences (CDSs), GC% content, and the TIRs regions) are presented in the genome maps in Figure 1.

### Determining the Closest Terrestrial Type-Strain Relative for the Marine Sponge-Derived Isolates

In order to analyze possible niche adaptations in the marine sponge-derived SM17 and SM18 isolates, phylogenetic and whole-genome alignment analyses were performed to identify the closest terrestrial type-strain relative, with the complete genome sequence available in GenBank, of each isolate; with a view to performing subsequent phenotypic, morphological and genomic comparisons once these relatives had been determined.

**FIGURE 2:**
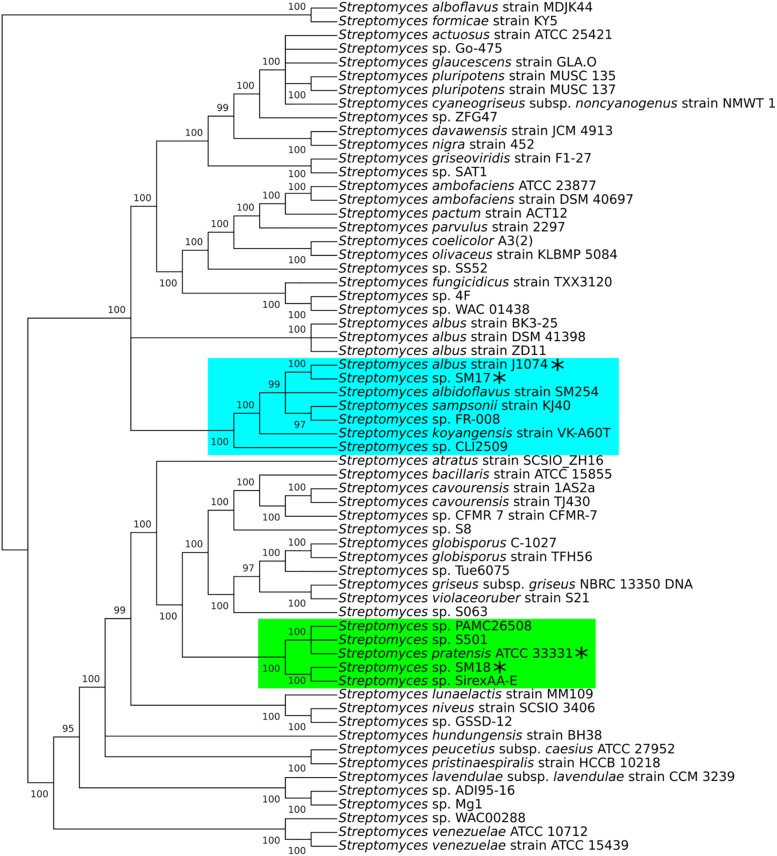
Phylogenetic tree of the concatenated nucleotide sequence of the 16S rRNA gene, plus the housekeeping genes *atpD*, *gyrB*, *recA*, *rpoB*, and *tyrB*. Including in this analysis are the SM17 and SM18 isolates, plus 60 *Streptomyces* isolates with complete genomes available in the GenBank database. Generated using MrBayes and MEGA X, with a posterior probability cut off of 95%.

Phylogenetic analysis was performed using the 16S rRNA and other housekeeping aforementioned genes, which allowed us to determine that *S. albus* J1074 and *S. pratensis* ATCC 33331 were the closest type-strain relative to the SM17 and SM18 strains, respectively (Figure 2). Notably, SM17 and J1074 – a derivative of the soil isolate *Streptomyces albus* G (Chater and Wilde, 1976, 1980) – are included in the same sub-clade, while SM18 and ATCC 33331 are not, indicating that the latter pair are more distantly related than the former. Nevertheless, further analyses were performed with the ATCC 33331 strain, as it was the type-strain included in the SM18 clade that was readily available in culture collections. Also, it is important to note that the ATCC 33331 strain is the only soil-derived isolate present in the SM18 clade (NCBI BioSample: SAMN00191232), while SirexAA-E was isolated from an insect/microbe symbiotic community (Bianchetti et al., 2013); PAMC26508 was isolated in association with the Antarctic lichen *Cladonia borealis* (Shin et al., 2013); and S501 was isolated from the sediment from a seaside wetland (NCBI BioSample: SAMN10144670). Thus, for these aforementioned reasons (being a type-strain with its complete genome available on GenBank, isolated from soil, and available from culture collections), the ATCC 33331 strain was determined to be the most suitable isolate identified in the SM18 clade for the purposes of this study.

To further support the similarities between our marine strains, SM17 and SM18, and their closest terrestrial counterparts, J1074 and ATCC 33331, alignments of the individual 16S rRNA and the other housekeeping genes were performed with NCBI BLASTN and BLASTX (Table 2). The high identity values determined by the analysis allowed further comparisons to be determined between the related pairs, and also between all four *Streptomyces* strains. Notably, the identities for the SM17- *S. albus* J1074 pair are higher (>99% for all the genes analyzed) than those for the SM18 – *S. pratensis* ATCC 33331 pair; (91% to 99% identity depending on the gene using BLASTN, and >95% using BLASTX). This further indicates that SM17 and *S. albus* J1074 are very closely related organisms – possibly even belonging to the same species, while the SM18 and *S. pratensis* ATCC 33331 are more distantly related.

**TABLE 2:** 16S rRNA and housekeeping gene alignment comparisons using the NCBI BLAST tool, between the pairs SM17 and *S. albus* J1074 (second column: SM17-J1074); and SM18 and *S. pratensis* ATCC 33331 (third column: SM18-ATCC 33331).

**Gene**	**SM17-J1074**	**SM18-ATCC 33331**
16S rRNA	1523/1524 (99%)	1519/1523 (99%)
*atpD* (ATP synthase subunit beta)	1442/1443 (99%) 480/480 (100%)	1395/1443 (97%) 454/480 (95%)
*gyrB* (DNA gyrase subunit B)	2123/2124 (99%) 706/707 (99%)	1943/2127 (91%) 683/708 (96%)
*recA* (recombinase RecA)	1123/1125 (99%) 333/333 (100%)	1058/1132 (93%) 326/332 (98%)
*rpoB* (DNA-directed RNA polymerase subunit beta)	3480/3483 (99%) 1142/1142 (100%)	3342/3487 (96%) 1116/1142 (98%)
*trpB* (tryptophan synthase beta chain)	1263/1263 (100%) 420/420 (100%)	1178/1281 (92%) 390/406 (96%)

*For the housekeeping genes, the first values presented are the BLASTN (nucleotide-nucleotide) alignment identities, while the second values below are the BLASTX (translated nucleotide-protein) alignment identities. For the 16S rRNA analysis only the BLASTN alignment was performed.*

Following the 16S rRNA and housekeeping genes analyses, *S. albus* J1074 and *S. pratensis* ATCC 33331 were selected for subsequent similarity analysis using a whole-genome alignment approach with the MUMmer program (Supplementary Figure S1). Large sections of the genomes are quite well conserved between the marine sponge-derived isolates and their closest relative organism, particularly when comparing SM17 with *S. albus* J1074 (Supplementary Figure S1A). This result further confirms previous analyses, and further supports *S. albus* J1074 and *S. pratensis* ATCC 33331 as suitable terrestrial relatives, for comparative purposes.

Interestingly, previous studies also reported *Streptomyces* spp. marine sponge-derived isolates that were determined to be closely related to *S. albus* J1074 (Ian et al., 2014; Iniyan et al., 2016; Almeida et al., 2018). Some of these strains, namely PVA 94-07; GBA 94-10; and *Streptomyces albus* ICN33; were isolated from completely different sample types and geographic locations than those of the current study. While SM17, which based on the aforementioned comparative analysis appears to be closely related to *S. albus* J1074, was isolated from the sponge *Haliclona simulans* from Kilkieran Bay (Galway, Ireland), at a depth of 15 m; the strains PVA 94-07 and GBA 94-10 were isolated from the sponges *Phakellia ventilabrum* and *Geodia barretti*, respectively; from the Tautra ridge (Trondheim fjord, Norway), at a depth of 121 m (Ian et al., 2014), while *Streptomyces albus* ICN33 was isolated from the sponge *Acanthella elongata*, from the Colachel coast (Kanyakumari District, Tamil Nadu), at an unspecified depth (Iniyan et al., 2016). This raises the possibility that “*albus*-like” *Streptomyces* strains may be ubiquitously associated with marine sponges.

### Phenotype, Morphology, and Differential Growth Assessment

**FIGURE 3:**
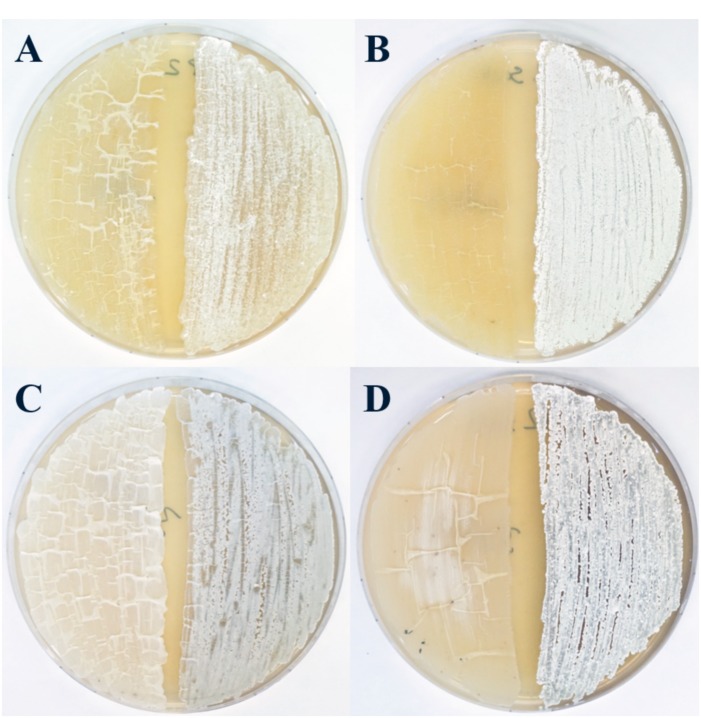
Differential growth assessment of marine and terrestrial *Streptomyces* strains. From left to right, **(A)** *S. albus* J1074 and SM17 on ISP2 agar medium; **(B)** *S. albus* J1074 and SM17 on ISP2 + ASW agar medium; **(C)** *S. pratensis* ATCC 33331 and SM18 on ISP2 agar medium; **(D)** *S. pratensis* ATCC 33331 and SM18 on ISP2 + ASW agar medium, following 3 days growth.

Members of the *Streptomyces* genus are known to be capable of colonizing a wide variety of different ecosystems, including soil, rhizosphere, lake and marine sediments, and have also been reported to be associated with insects, lichen, and sponges (Goodfellow and Fiedler, 2010; Bianchetti et al., 2013; Rashad et al., 2015; Liu et al., 2017; Ay et al., 2018; Jackson et al., 2018). Thus, it is reasonable to assume that these organisms possess a genetic plasticity and capability that facilitates their adaptation to such varied environmental niches (Hoff et al., 2018). Interestingly, previous studies have reported that *Streptomyces* spp. isolated from marine environments often possess the capacity of growing independently of the presence of sea salts in the growth medium (Goodfellow and Fiedler, 2010; Ian et al., 2014). In fact, many marine isolates often display very active metabolic profiles under such conditions (Goodfellow and Fiedler, 2010). To assess whether the SM17 and SM18 isolates had phenotypical and/or morphological differences with respect to their ability to grow under different conditions, they were cultured in ISP2 medium with and without the presence of ASW and compared with their terrestrial relatives (Figure 3), in a similar fashion to work previously conducted by the Zotchev group (Ian et al., 2014).

All the pair-wise comparisons showed clear morphological differences between the marine sponge-derived isolates and their respective terrestrial counterparts (Figure 3). All the isolates grew effectively in the ISP2 medium without ASW (Figures 3A,C), even though there were slight differences regarding growth and sporulation; with the SM17 isolate being able to grow and sporulate more rapidly in comparison to *S. albus* J1074 (Figure 3A). There was no clear difference in the growth of SM18 and *S. pratensis* ATCC 33331 on the ISP2 growth medium without ASW, although they clearly displayed very different morphological features (Figure 3C). On the other hand, when grown on the ISP2 medium with ASW, *S. albus* J1074 was clearly less capable of growing in the presence of sea salts, while SM17 thrived (Figure 3B). This result is particularly interesting, since, as previously shown (Figure 2, Table 2, and Supplementary Figure S1), these two organisms are genetically very similar. In contrast, there were less marked differences in the ability of both SM18 and *S. pratensis* ATCC 33331 to grow in the presence of sea salts (Figure 3D). While SM18 appeared to grow better, nevertheless *S. pratensis* ATCC 33331 was still able to grow in the ISP2 medium containing sea salts albeit more slowly than SM18; and indeed, more slowly than when *S. pratensis* ATCC 33331 was cultured in the absence of ASW (Figure 3C). From these observations, it became clear that a more thorough analysis of the SM17 and SM18 genomes might provide some interesting insights regarding potential genome-wide adaptations that may have occurred in these marine isolates, which may have resulted in them being able to grow more efficiently in the ISP2 medium supplemented with ASW; relative to their terrestrial counterparts.

### Prediction of Secondary Metabolite Biosynthetic Gene Clusters (smBGCs)

Members of the Actinomycetales order are historically known to produce a broad range of bioactive compounds of biotechnological and clinical interest, and among them, the *Streptomyces* genus excels, with over 10,000 bioactive compounds produced by members of the genus being discovered to date (Hwang et al., 2014; Ziemert et al., 2016; Kamjam et al., 2017; Lee et al., 2018). The marine sponge-derived SM17 and SM18 strains have previously been reported to possess antimicrobial activity against gram-negative and gram-positive bacteria – including the methicillin-resistant *S. aureus* (MRSA), and yeasts (Kennedy et al., 2009; Jackson et al., 2018). To provide insights at a genomic level regarding which compounds might be responsible for the previously observed antimicrobial activity, we employed the antiSMASH program in an attempt to predict the presence of putative smBGCs, based on homology to known smBGCs deposited in the databases (Blin et al., 2017). Several gene clusters were predicted to be present in both SM17 and SM18 (Supplementary Tables S1, S2), with a total of 20 potential smBGCs in SM17, and 26 in SM18; with a variety of cluster types being assigned, including: type I polyketide synthases (T1pks), type II polyketide synthases (T2pks), type III polyketide synthases (T3pks), non-ribosomal peptide synthetases (NRPS), lantipeptides, bacteriocins, and terpenes. These types of clusters are known to produce a variety of compounds with antimicrobial activity, including: erythromycin (T1pks); tetracenomycin (T2pks); germicidin (T3pks); daptomycin (NRPS); nisin (lantipeptide/lantibiotic/bacteriocin); and pentalenolactone (terpene) (Shen, 2003; Tetzlaff et al., 2006; Robbel and Marahiel, 2010; Shi et al., 2011; Yamada et al., 2015; Čihák et al., 2017).

**FIGURE 4:**
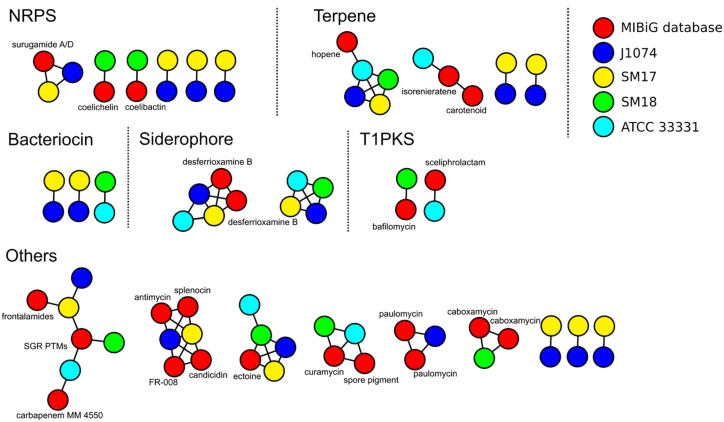
Gene clusters families (GCFs) analysis using antiSMASH (version 4), BiG-SCAPE (version 20181005), MIBiG database (version 1.4), and CytoScape. Each node represents a smBGC predicted in the respective organism (labeled in different colors), and the interactions represent cluster similarity. Annotations of the MIBiG database smBGCs are labeled accordingly. Singletons, i.e., smBGCs without similarities with the smBGCs in the MIBiG database, or without similarities with the smBGCs predicted in the other genomes analyzed in this study, are not included in this figure.

The antiSMASH predictions were also further analyzed using the BiG-SCAPE program (Navarro-Muñoz et al., 2018), which allowed us to cluster the predicted smBGCs into gene cluster families (GCFs) based on their sequences and Pfam protein families similarities (El-Gebali et al., 2019), and also to compare them to known smBGCs available from the latest version of the MIBiG repository (version 1.4) (Medema et al., 2015), which can also assist in improving the annotations of the predicted smBGCs. Based on their similarity to known smBGCs, some of the bioactive compounds predicted to be encoded by these smBCGs may be compatible with the previously determined antimicrobial capabilities of the SM17 and SM18 isolates (Figure 4 and Supplementary Tables S1, S2). For example, SM17 appears to possess a candicidin, an antimycin, and a polycyclic tetramate macrolactam cluster (SGR PTMs) (Figure 4 and Supplementary Table S1), with similarity to the candicidin, antimycin and tetramate macrolactam sequences from *Streptomyces* sp. FR-008, *Streptomyces* sp. S4 and *Streptomyces griseus* in the database, respectively, and which are known to have anti-fungal properties (Campelo and Gil, 2002; Chen et al., 2003; Seipke et al., 2011; Luo et al., 2013). SM17 also contains clusters that may potentially encode for the production of surugamides (Figure 4) and the glycopeptide antibiotic mannopeptimycin (Supplementary Table S1), with the former possessing gene similarity with the surugamide A/D sequence from *Streptomyces albus* in the database (Ninomiya et al., 2016), and the latter sharing similarity to the mannopeptimycin sequence from *Streptomyces hygroscopicus* in the database, with the main biosynthetic genes being present in the predicted smBGC (Singh et al., 2003; Magarvey et al., 2006). SM18 appears to possess a cluster encoding the anti-bacterial compound bafilomycin (Figure 4 and Supplementary Table S2), with similarity to the bafilomycin sequence from *Streptomyces lohii* (Bowman et al., 1988; Zhang et al., 2013; Nara et al., 2017); as well other clusters with similarity to known smBGCs that encode anti-fungal and anti-bacterial compounds such as SGR PTMs, curamycin, and caboxamycin (Figure 4), from *Streptomyces griseus* (Luo et al., 2013), *Streptomyces curacoi* (Galmarini and Deulofeu, 1961), and *Streptomyces* sp. NTK 937 (Hohmann et al., 2009; Losada et al., 2017), respectively.

**FIGURE 5:**
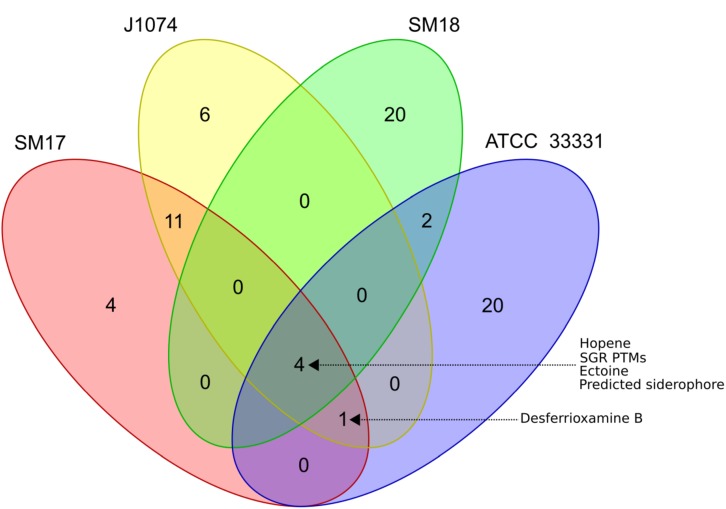
Venn diagram representation of GCFs presence/absence analysis using BiG-SCAPE.

We also performed the antiSMASH and BiG-SCAPE analysis on *S. albus* J1074 and *S. pratensis* ATCC 33331 genomes, in an effort to determine to what extent the marine sponge-derived isolates SM17 and SM18 may potentially produce similar and/or unique compounds when compared to their terrestrial counterparts (Figure 4 and Supplementary Tables S3, S4). Based on the BiG-SCAPE similarity clustering, a Venn diagram was generated, representing the presence/absence of GCFs in the SM17, SM18, *S. albus* J1074, and *S. pratensis* ATCC 33331 genomes (Figure 5). In keeping with the phylogeny results which indicated that SM17 and *S. albus* J1074 were very closely related organisms, the smBGCs predictions and similarity clustering results were also strikingly similar (Figures 4, 5). Among a total of 42 predicted smBGCs in both genomes (22 in *S. albus* J1074 and 20 in SM17), 10 seem to be unique (6 in *S. albus* J1074, and 4 in SM17) (Figure 5). In contrast, there was a much larger number of predicted unique smBGCs between SM18 and *S. pratensis* ATCC 33331, where amongst a total of 53 predicted clusters (27 in *S. pratensis* ATCC 33331 and 26 in SM18), only 6 appear to be present in both genomes; with the majority being potentially unique (20 in *S. pratensis* ATCC 33331 and 20 in SM18) (Figure 5). Also, a total of 4 smBGCs were shared among all of the strains analyzed (Figure 5), and these were determined to be: hopene; SGR PTMs family of smBGCs, ectoine, and a predicted siderophore smBGC without significant similarity to sequences in the MIBiG database (Figures 4, 5).

Notably, smBGCs encoding the production of desferri- oxamines, which are hydroxamate siderophores, while present in *S. albus* J1074, *S. pratensis* ATCC 33331 and SM17 (Figures 4, 5), are absent in the SM18 genome (Supplementary Table S2 and Figure 4). Siderophores are specialized metabolites that function to scavenge Fe^3 +^ , and hence are crucial for sessile organisms to assimilate iron (Hider and Kong, 2010). Genes involved in desferrioxamines production, in particular, are widely conserved in marine microorganisms, and are believed to be present in all *Streptomyces* species (Tierrafría et al., 2011; Cruz-Morales et al., 2017). Thus, this may be the first report of a *Streptomyces* isolate that does not possess a smBGC that encodes for the production of desferrioxamines. The SM18 isolate does, however, possess smBGCs encoding other siderophores, such as coelichelin, and mirubactin (Supplementary Table S2 and Figure 4), which may circumvent for the lack of production of desferrioxamines with respect to iron acquisition in the strain.

During processing of the data for this study, a newer version of the antiSMASH webserver (version 5) was released (Blin et al., 2019). Using this new version of antiSMASH did not result in any major differences being detected in the data being analyzed, it did however result in the identification of a smBGC encoding mycemycin in the SM18 genome. Mycemycin is a relatively newly identified compound, from marine and soil *Streptomyces* isolates, belonging to the dibenzoxazepinone (DBP) family, which possesses HIV-1 reverse transcriptase inhibitory activity (Liu et al., 2015; Song et al., 2018). Production of the DBP family of compounds appear to date to be rare in the microbial world, and these compounds possess a broad range of interesting activities, including anti-HIV and anti-tumor activities (Zhang et al., 2018). Thus, pursuing the identification of new members of this family of compounds may be worthwhile, and it is interesting to report the potential presence of a smBGC encoding the production of mycemycin in another *Streptomyces* isolate.

Nevertheless, it is clear that further analysis would need to be undertaken to confirm that these compounds are in fact being produced by the SM17 and SM18 isolates, as some of these smBGCs are likely to be cryptic and the compounds may not be produced under certain culture conditions (Rutledge and Challis, 2015; Rigali et al., 2018). Given that SM17 and *S. albus* J1074 are genetically very similar, it is perhaps reasonable to expect that regulation of secondary metabolite production may to some extent be similar in both strains. Therefore, it may be possible to use what is currently known about the better studied *S. albus* J1074 isolate to gain a better understanding regarding the expression of certain smBGCs and the metabolic pathways involved in SM17 (Hoz et al., 2017; Kallifidas et al., 2018; Nguyen et al., 2018).

### Comparative Genomics

A series of comparative genomics analyses was then performed in order to further characterize the marine sponge-derived isolates SM17 and SM18 at the genome level, and in particular to compare them to their respective closest terrestrial relative.

#### Analysis of Orthologous Genes

**FIGURE 6:**
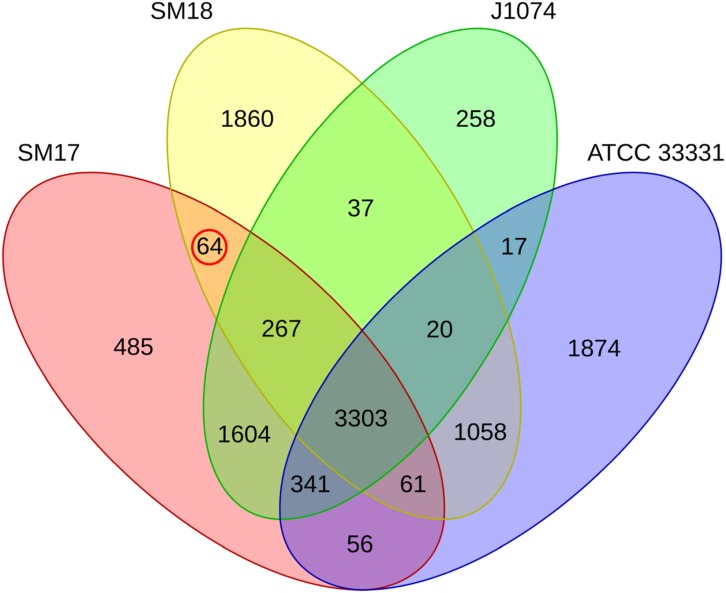
Venn diagram representing the presence/absence of orthologous genes in the SM17, SM18, *S. albus* J1074, and the *S. pratensis* ATCC 33331 genomes. Orthologous genes that are present commonly in the marine sponge-derived isolates SM17 and SM18, while absent in their terrestrial counterparts J1074 and ATCC 33331, are circled in red.

The Roary program was used to determine the pan-genome; the core genome; the accessory genome; and the strain-specific genome (the genes that are uniquely present in only one of the isolates), in the marine sponge-derived isolates *Streptomyces* sp. strain SM17 and *Streptomyces* sp. strain SM18, and their respective closest terrestrial relatives *S. albus* J1074 and *S. pratensis* ATCCC 33331 (Figure 6) (Page et al., 2015). The pan-genome was determined to consist of 11,305 genes; while the core genome consisted of 3,303 genes (∼29% of the pan-genome); and the accessory genome consisted of 8,002 genes (∼71% of the pan-genome). For the strain-specific genomes, SM17 had 485 unique genes; SM18 had 1,860 unique genes; *S. albus* J1074 had 258 unique genes; and *S. pratensis* ATCC 33331 had 1,874 unique genes. This is a combined total of 4,477 unique genes (∼39% of the pan-genome, and ∼56% of the accessory genome). Notably, in keeping with what we had previously observed with the phylogeny and whole-genome alignment analyses, the SM17 and J1074 strains shared a very large number of orthologous genes (a total of 5,515 shared genes, or ∼89% and ∼94% of the SM17 and the J1074 total number of CDSs, respectively), further indicating that they are very closely related organisms. In contrast, SM18 and *S. pratensis* ATCC 33331 shared a much lower proportion of their genes: 4,469 genes (or ∼67% and ∼66% for the SM18 and ATCC 33331 total number of CDSs, respectively). A total of 64 orthologous genes were found to be commonly present in the marine sponge-derived isolates SM17 and SM18, while absent in their terrestrial counterparts J1074 and ATCC 33331 (Figure 6). Given that they are absent in both terrestrial relatives, we undertook further analyses of these genes to assess their potential function(s) in an effort to provide insights into potential ENAs in both these sponge-derived isolates.

#### Orthology Analysis of smBGC-Associated Genes

The genes previously determined to be associated with smBGCs, using the antiSMASH program, were subsequently analyzed using the Roary program, to identify smBGCs-associated genes which were shared or unique between the four genomes (Supplementary Figure S2) (Page et al., 2015; Blin et al., 2017). With respect to potential smBGCs-associated genes, very few genes appeared to be conserved in all the organisms (a total of 58 genes, corresponding to 2.8% of the total smBGCs-associated gene pool, or 0.017% of the core genome) (Supplementary Figure S2). The largest number of unique smBGCs-associated genes is present in the SM18 isolate (623 genes), followed by *S. pratensis* ATCC 33331 (485 genes) which may be indicative of a greater potential to produce diverse secondary metabolites in these isolates. In contrast, SM17 and *S. albus* J1074 appear to possess a lower quantity of unique smBGCs-associated genes; with 132 and 150 unique genes, respectively (Supplementary Figure S2).

Interestingly, when comparing the Venn diagrams from Figure 6 and Supplementary Figure S2, it appears that a large portion of the unique genes present in the isolates are potentially related to the production of secondary metabolites. For SM17, ∼27% (132 out of 485) of the unique genes are potentially smBGCs-associated genes, while for SM18 this percentage is ∼33% (623 out of 1860); ∼58% (150 out of 258) for *S. albus* J1074; and ∼26% (485 out of 1874) for *S. pratensis* ATCC 33331. Taken together, these results indicate that, even for closely related *Streptomyces* spp. isolates (particularly when considering the pair SM17 and *S. albus* J1074), there is still potential to discover different secondary metabolites from these strains, with potentially unique characteristics. Previous reports have also indicated that the use of closely related *Streptomyces* strains to identify new smBGCs is useful for the identification of novel specialized biosynthetic pathways (Antony-Babu et al., 2017; Vicente et al., 2018).

#### Groups of Orthologous Genes Commonly Present in the Marine Sponge-Derived Isolates

Given that the SM17 and SM18 were isolated from a marine sponge and have been shown to be more adapted to higher salinity medium (Figure 3), it is likely that the identification of genes that are commonly present in SM17 and SM18 but not in their terrestrial relatives J1074 and ATCC 33331 may help in the identification of potential ENAs that these strains might possess, at a genetic level. The previous analysis of orthologous genes allowed us to determine which groups of orthologous genes are present commonly in the marine sponge-derived isolates SM17 and SM18, while absent in their terrestrial counterparts J1074 and ATCC 33331, as highlighted in Figure 6. This was performed by taking into account sequence homology and gene synteny (e.g., splitting paralogous genes with the Roary program); hence different copies of a gene can belong to different orthologous group due to potentially different evolutionary events such as gene duplication or lateral gene transfer occurring in the *Streptomyces* genomes (Zhou et al., 2012; Page et al., 2015). Thus, from here on the orthologous genes will be referred to simply as “genes.” In doing this we identified a potential ENA gene pool which consisted of 64 genes (Table 3). These were then manually annotated using the NCBI GenBank, CDD, UniProt, and the InterPro databases (Johnson et al., 2008; Marchler-Bauer et al., 2011; UniProt Consortium, 2015; Benson et al., 2018; Mitchell et al., 2018), and hypothetical proteins were removed, resulting in a final total of 57 genes (Supplementary Table S5). The ENA gene pool included functional categories of genes that are likely to be related to niche adaptations in the marine sponge-derived isolates, and included a total of 29 genes that could be grouped based on potential biological functions such as osmotic stress, defense; transcriptional regulation; symbiotic interactions; antimicrobial compounds production and resistance; ABC transporters; together with horizontal gene transfer and defense-related features (Table 3).

**TABLE 3:** Groups of orthologous genes and their respective annotations (excluding hypothetical proteins), which are present commonly in the sponge-derived isolates SM17 and SM18, while absent in their terrestrial counterparts J1074 and ATCC 33331.

**Environmental niche adaptation**	**Gene name**	**Product**
Osmotic stress defense	*nuoA*	NADH-quinone oxidoreductase subunit A
	*nuoH*	NADH-quinone oxidoreductase subunit H
	*nuoJ*	NADH-quinone oxidoreductase subunit J
	*nuoK*	NADH-quinone oxidoreductase subunit K
	*nuoL*	NADH-quinone oxidoreductase subunit L
	*nuoM*	NADH-quinone oxidoreductase subunit M
	*nuoN*	NADH-quinone oxidoreductase subunit N
	*proP*	Proline/betaine transporter
Transcriptional regulation	bepR^*^	HTH-type transcriptional repressor BepR / TetR family transcriptional regulator
	*cynR*	HTH-type transcriptional regulator CynR / LysR family transcriptional regulator
	*degU*	Transcriptional regulatory protein DegU / DNA-binding response regulator
	group_5796	Transcriptional regulator, IclR family
	group_5819	Transcriptional regulator PadR-like family protein
	*rhmR*	HTH-type transcriptional regulator KipR / MarR family transcriptional regulator
	*tcrA*	Transcriptional regulatory protein CutR / DNA-binding response regulator
Symbiotic interactions	group_5772	Tetratricopeptide repeat protein
Antimicrobial compounds production and resistance	aprX^*^	Serine protease AprX / Subtilase family protein / Peptidase S8
	group_5198	Aminoglycoside phosphotransferase
	group_5385	Aminoglycoside phosphotransferase
	group_5818	Acyltransferase 3
	group_5836	Acyltransferase
	*liaS*	HPK7 family sensor histidine kinase LiaS
ABC transporters	group_5821	ABC transporter permease
	*tauB*	Aliphatic sulfonates import ATP-binding protein SsuB/ABC transporter ATP-binding protein
	*yknY*	Uncharacterized ABC transporter ATP-binding protein YknY
Horizontal gene transfer and defense-related features	group_1044	Integrase core domain/IS3 family transposase
	group_1272	Toxin-antitoxin system, RelE family
	group_1944	Restriction endonuclease
	group_1945	IS3 family transposase

When the gene name was not determined, a generic unique name was given (e.g., group_5796) by the Roary program. ^*^genes without multiple copies or paralogs in the terrestrial isolates’ genomes, taking into consideration only those with a defined gene name.

##### Resistance to osmotic stress

**FIGURE 7:**
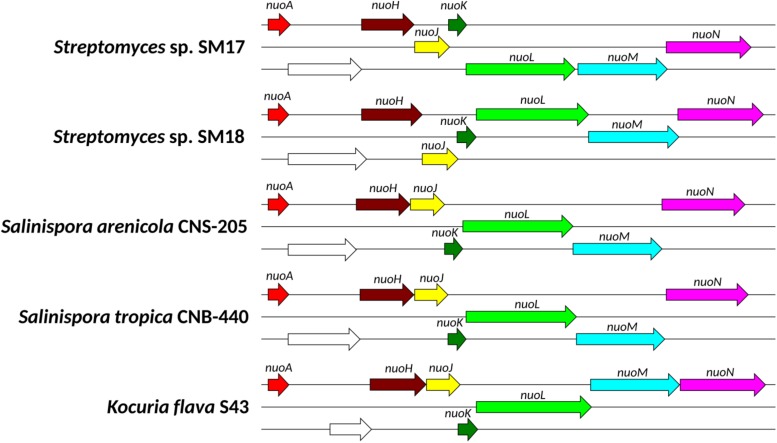
Graphical representation of the gene synteny of the partial *nuo*-operon present in the genomes of the marine isolates *Streptomyces* sp. SM17, *Streptomyces* sp. SM18, *Salinispora arenicola* CNS-205, *Salinispora tropica* CNB-440, and *Kocuria flava* S43, while absent in the terrestrial isolates *Streptomyces albus* J1074 and *Streptomyces pratensis* ATCC 33331. Each of the three lines represent a reading frame and the arrows represent a gene, with their respective gene names. Genes with the same color are homologs, while the ones in white are hypothetical proteins with no homologs in the UniProt or PDB databases.

For bacteria to survive in marine environments where salinity levels of approximately 3.5% exist, they must be able to simultaneously overcome stresses due to both high osmotic pressure and high Na + concentrations (Yaakop et al., 2016); together with other stresses including pressure, temperature and oligotrophic conditions (Xie et al., 2018). Bacteria typically respond to variations in external osmotic pressure by accumulating or releasing solutes, thereby attenuating water fluxes and maintaining cellular homeostasis (Wood, 2015). The marine sponge-derived isolates SM17 and SM18 appeared to grow and differentiate more rapidly when grown on media containing artificial seawater, when compared to their closely related terrestrial counterparts (Figure 3), thus indicating a potential increased fitness to higher salinity environments, as also previously described in other marine *Streptomyces* isolates (Ian et al., 2014). Previous studies with marine Actinomycetes, specifically with the genera *Salinispora*, *Streptomyces*, and *Kocuria*, have proposed that the NADH-quinone oxidoreductases *nuoAHJKLMN* genes, which encode a proton pump, could be classified as potential MAGs (Penn and Jensen, 2012; Ian et al., 2014; Sun et al., 2018). This proton pump is believed to create a proton-motive force which generates ATP, helping to maintain a proton gradient in seawater (Penn and Jensen, 2012; Ian et al., 2014; Sun et al., 2018). We identified the *nuoAHJKLMN* genes in the ENA gene pool in both SM17 and SM18 (Table 3). Further analysis indicated that both isolates possessed one extra copy of these genes when compared to their terrestrial counterparts, and that these genes were organized in an operon-like structure, similar to that previously reported in *Salinispora arenicola* CNS-205 and in *Kocuria flava* S43 (Sun et al., 2018). Furthermore, the same gene synteny for the partial *nuo*-operon was present in *Streptomyces* sp. SM17, *Streptomyces* sp. SM18, *Salinispora arenicola* CNS-205, *Salinispora tropica* CNB-440, and *Kocuria flava* S43 (Figure 7); with *nuoA*, followed by a hypothetical protein, and then followed by *nuoH*, *nuoJ*, *nuoK*, *nuoL*, *nuoM*, and *nuoN*. It is important, however, to note that differences in sequence identity and reading frames are present (Supplementary Table S6 and Figure 7), which may indicate that different evolutionary events may have occurred in the aforementioned genomes. The presence of this partial *nuo*-operon in the sponge derived SM17 and SM18 isolates and in the other marine actinomycetes (*Salinispora arenicola* CNS-205, *Salinispora tropica* CNB-440, and *Kocuria flava* S43), which are absent in their terrestrial counterparts J1074 and ATCC 33331, may explain, at least in part, the increased tolerance to salinity we observed in SM17 and SM18 relative to J1074 and ATCC 33331; which although still able to grow in the presence of ASW, grew much more slowly (Figure 3). Another important mechanism which bacteria employ as a defense mechanism against osmotic stress is both the synthesis and the uptake of compatible solutes, such as proline, glycine, betaine and ectoine, in order to maintain membrane turgor pressure (Krämer, 2010; Lim and Lee, 2015). Extra copies of the *proP* gene, which encodes a potential proline/betaine transporter (ProP), were found in both the SM17 and SM18 strains. It has been shown in *E. coli* that the ProP transporter acts both as an osmoregulator and as an osmosensor; and is capable of transporting proline, glycine betaine, proline betaine, carnitine, ectoine and other compounds (MacMillan et al., 1999; Roessler and Muller, 2001; Burg and Ferraris, 2008). Therefore, the *proP* genes may also be related to the increased capacity of the SM17 and SM18 strains to tolerate hyperosmotic environments, as evidenced by their growth on the ASW medium (Figure 3).

##### Antimicrobial compounds production and resistance

For many years the main ecological function of antibiotics production in bacteria in natural environments was believed to be inhibition of the growth of other microorganisms, thereby conferring a selective advantage on the producing strain with respect to colonization of particular environmental niches (Linares et al., 2006). In this respect, antibiotic production may be employed as a defense mechanism for the *Streptomyces* spp. isolates SM17 and SM18 – and other members of the symbiotic community – against other competitor microorganisms in the marine sponge host; as has been previously reported to be the case with other antibiotic producing microorganisms, such as *Streptomyces* spp. which have been isolated from different hosts including plants and insects (Bondarev et al., 2013; van der Meij et al., 2017; Ceapã et al., 2018; Engl et al., 2018). Furthermore, antibiotics may also play an important role in the overall defense of the sponge host itself by protecting it against pathogens, in a biological interaction defined as defensive symbiosis (Clay, 2014), which has been reported in a number of systems, including beewolf wasps and antibiotic-producing *Streptomyces* bacteria (Engl et al., 2018). Nevertheless, more recently it has been proposed that in natural environments antibiotics may also act as small molecules with signaling functions, functioning in a similar fashion to quorum sensing molecules; acting for example to alter the expression of genes; to induce biofilm formation; or to modulate colony morphology – all of which may be important in coordinated communication within symbiotic communities (Romero et al., 2011). Thus, antibiotics may have a number of roles in a niche environment such as in marine sponges, which may include both defense-related and signaling roles (Linares et al., 2006; Romero et al., 2011). The presence of a wide variety of predicted smBGCs in both SM17 and SM18 – many of which are potentially involved in the production of antimicrobial compounds (Supplementary Tables S1, S2), coupled with the previously reported antimicrobial activities in these strains (Kennedy et al., 2009; Jackson et al., 2018); supports a possible role for these two *Streptomyces* spp. isolates in defensive symbiosis in *Haliclona simulans*, from which they were isolated. In this respect two acyltransferase genes potentially involved in the biosynthesis of type II PKS antibiotics, or type I PKSs that require discrete acyltransferase enzymes, were present in the ENA gene pool (Table 3) (Cheng et al., 2003; Zhang et al., 2017). In addition, a subtilase-like serine protease gene (*aprX*) was also identified in the ENA gene pool (Table 3), which belongs to a family of proteins that are known to play a number of different biological roles, including involvement in the biosynthesis of antimicrobial peptides, with some possessing algicidal properties, which could potentially be relevant from a sponge defense perspective (Lee et al., 2000; Barra et al., 2017; Montalbán-López et al., 2018). Protease producing marine bacteria are known to be important in the degradation of organic nitrogen which is essential for nitrogen recycling in marine sediments (Zhang et al., 2015). Marine bacterial proteases are also known to play a role in sponge host nitrogen metabolism, which may also explain the presence of this specific protease in the marine isolates SM17 and SM18, and its absence in their terrestrial counterparts (Li et al., 2016; Kiran et al., 2018). Further work on these specific proteases might also be relevant from an industrial perspective, given the interest in proteases of marine origin which are typically cold adapted, salt tolerant, with broad optimal pH values (Li et al., 2016) and which are particularly suited for a number of biotechnological applications, including laundry detergents, food processing, the leather and textile industries, and in waste water treatment applications (Li et al., 2013; Salwan and Sharma, 2018).

A LiaS-encoding gene was also present in the ENA pool, which has previously been reported to be part of the two-component LiaS/LiaR regulatory system, a stress-sensing module that is conserved in *Firmicutes* bacteria and which is involved in the response to a subset of cell wall-active antibiotics such as bacitracin and vancomycin in *Bacillus subtilis*; while also being involved in response to cationic antimicrobial peptides and secretion stress (Mascher et al., 2004). In *Listeria monocytogenes*, the LiaS/R system also plays an important role in resistance to the food preservative nisin (Collins et al., 2012). The presence of antibiotic resistance-related genes in our two sponge-derived isolates may be significant from two perspectives. Firstly, they may function as part of a self-resistance mechanism in these strains, allowing them to be protected from the antimicrobial compounds that they themselves are producing; and/or secondly, as a resistance mechanism to protect themselves from the antimicrobial compounds produced by other microorganisms within the sponge symbiotic community (Wright, 2005, 2012).

##### ABC transporters

ATP-binding cassette (ABC) transporters are ATP-dependent protein complexes that are widespread in all forms of life and which are vital in mediating the transport of both organic and inorganic molecules across cell membranes (ter Beek et al., 2014; Wilkens, 2015). In bacteria, they confer resistance to antibiotics and to other toxic compounds through efflux/transport mechanisms (Greene et al., 2018); and are also involved in nutrient acquisition and in helping to maintain osmotic balance in the cell (Wood, 2007; Fan et al., 2013; Teichmann et al., 2018). The ENA gene pool includes an *yknY*-like ABC transporter (Table 3), which has been reported to be involved in the efflux of the sporulation-delaying protein (SDP) in *Bacillus* spp., although it is still poorly characterized in other genera, such as in *Streptomyces* (González-Pastor et al., 2003; Xu et al., 2016; Greene et al., 2018). The SDP protein is a killing factor exported by cells that have started the sporulation process, therefore inducing the lysis of sister cells, making more nutrients available, and ultimately delaying the sporulation process and maintaining regular cell growth (González-Pastor et al., 2003). Thus, it is reasonable to assume that the bacterial members of the sponge symbiotic community may employ similar mechanisms and resistance genes targeting these potentially harmful proteins, which may be the case in both SM17 and SM18. A *tauB*/*ssuB*-like ABC transporter was also present in the ENA gene pool, which may be responsible in allowing more versatile nutrient acquisition and cycling – specifically for nitrate and sulfonate – for the marine *Streptomyces* isolates SM17 and SM18, as it has been previously suggested to be the case for marine sponge symbiotic communities, through metagenome binning analysis (Karimi et al., 2018).

##### Transcriptional regulation

Being able to efficiently respond to changes in their environment is crucial in helping bacteria adapt to and survive within these environments (Feklístov et al., 2014; Daniel-Ivad et al., 2018); and, as previously mentioned, it is particularly important for the sponge-derived bacteria to be able to react appropriately to osmotic and other environmental stresses such as the presence of antibiotics and other potentially harmful compounds; the lack of nutrients; or allowing cell-to-cell communication through quorum sensing.

Transcriptional regulators play a crucial role in allowing bacteria to respond appropriately to numerous environmental stimuli and are believed to be intrinsically linked to lifestyle and environmental adaptation in bacteria (Stock et al., 1990; Feklístov et al., 2014; Daniel-Ivad et al., 2018). Since the SM17 and SM18 isolates inhabit the same niche environment and are subsequently exposed to similar conditions, it is likely that they employ similar adaptive mechanisms in response to those conditions. The ENA gene pool does include a range of transcriptional regulators (Table 3), further indicating that the marine sponge-derived isolates SM17 and SM18 share signal transduction mechanisms that are absent in their terrestrial counterparts, which may account for important niche adaptations that have been acquired. Notably, the TetR, LysR, DegU, IclR, PadR, and CutR families of transcriptional regulators are present in the ENA gene pool. These are commonly associated with mechanisms that could also be potential adaptations employed by the sponge-derived isolates SM17 and SM18, such as for example: antibiotics production (TetR, DegU); antibiotics resistance (TetR, LysR); multidrug resistance (IclR, PadR), quorum sensing (TetR, LysR, IclR); sporulation (IclR); detoxification (PadR); salt stress response (DegU); and copper stress response (CutR) (Huillet et al., 2006; Molina-Henares et al., 2006; Maddocks and Oyston, 2008; Fibriansah et al., 2012; Rademacher and Masepohl, 2012; Cuthbertson and Nodwell, 2013; Rodríguez et al., 2013; Tian et al., 2014; Hoffmann and Bremer, 2016).

For example, a gene encoding a LysR family transcriptional regulator, that is present uniquely in the SM18 genome, is located upstream of a gene which appears to encode a Beta-lactamase enzyme family protein, which are enzymes that provide mechanisms of resistance to β-lactam antibiotics (Majiduddin et al., 2002; Naas et al., 2017). In addition, a gene encoding a IclR family transcriptional regulator that is present in both the SM17 and SM18 genomes, is located upstream of a *proP* gene, which potentially encodes a proline/betaine transporter and, which previously mentioned, could be related to osmotic regulation in these organisms (MacMillan et al., 1999; Roessler and Muller, 2001; Burg and Ferraris, 2008). While a gene encoding a transcriptional regulator PadR-like family protein which is present in both the SM17 and SM18 genomes; is located upstream of a gene coding an ABC transporter, which as previously mentioned, could be involved with nutrient acquisition, resistance to toxic molecules, or in maintaining osmotic balance in these isolates (Wood, 2007; Fan et al., 2013; Greene et al., 2018; Teichmann et al., 2018).

##### Genomic evolution through horizontal gene transfer

Horizontal gene transfer (HGT) is an important mechanism in bacterial genome evolution, and commonly involves the acquisition of mobile genetic elements (MGEs) (Bellanger et al., 2014). Previous studies have reported that the genomes of symbiotic bacteria – including sponge symbionts – possess a higher number of MGEs than those of free-living microorganisms (Thomas et al., 2010; Fan et al., 2012, 2013). It has been proposed that MGEs play a crucial role in co-evolution with the host and convergent evolution of marine sponge symbiotic communities in a number of ways, such as enabling the members of the symbiotic community to share important traits for niche adaptation (Fan et al., 2012), such as for example genes related to stress tolerance, antibiotics resistance, and nutrient acquisition. In addition, the MGEs can function in the deactivation or removal of non-essential genes, such as those that are only required by free-living bacteria, or those related to functions that are already being performed by other members of the symbiotic community (Fan et al., 2012). Two genes encoding transposases were found in the ENA gene pool (Table 3), indicating that they may be involved in HGT events and co-evolution between the marine sponge isolates SM17 and SM18. Also, the three plasmids that were identified in SM17 (Table 1B and Figure 1), which are absent in its terrestrial counterpart *S. albus* J1074, provide additional evidence of potential genomic evolution through transferable elements occurring within the marine sponge microbiota.

The high filter feeding rates of sponges mean that they are likely to be exposed to phage attack from the plankton, and that bacterial sponge symbionts may be subjected to phage-mediated transduction which can lead to cell lysis (Thomas et al., 2010). Therefore, it might be expected that sponge bacterial symbiotic communities would require defense mechanisms to protect themselves from foreign DNA, such as restriction modification (R-M) systems and toxin-antitoxin (T-A) systems (Fan et al., 2012; Horn et al., 2016; Slaby et al., 2017). R-M systems are also linked to MGEs in that they can be transferred via the MGEs, or they can act as MGEs in transposon-like structures (Furuta and Kobayashi, 2013). In the ENA gene pool, we identified one restriction endonuclease that could be part of a transferrable R-M system and one T-A system gene from the RelE family in SM17 and SM18 (Table 3). This further highlights the possibility that HGT events may be occurring between the sponge-derived isolates and the possibility of shared niche adaptations between them, and also the requirement for defense mechanisms against foreign DNA in the symbiotic bacteria. Importantly, T-A systems have also been proposed to provide mechanisms to cope with stress – such as nutrient stress – by either programmed cell death or by inducing bacteriostasis, which may be another important role played by the T-A systems in symbiotic communities in oligotrophic environments (Van Melderen, 2010; de Goeij et al., 2013).

##### Eukaryotic-like proteins and potential host interaction

Metagenomic and genomic studies have reported that bacterial symbionts contain a large number of genes encoding for eukaryotic-like proteins (ELPs) (Reynolds and Thomas, 2016). ELPs contain repeat domains that are commonly found in eukaryotic proteins, such as tetratricopeptide repeats (TPRs), and are believed to play an important role in symbiotic relationships, by mediating protein-protein interactions for a range of cellular proteins (Thomas et al., 2010; Li et al., 2015; Reynolds and Thomas, 2016). These ELPs may have a broader function in mediating bacterial-sponge interactions and may modulate the host’s behavior (Li et al., 2015; Reynolds and Thomas, 2016). The ENA gene pool contained a tetratricopeptide repeat-containing protein, which is a class of ELP that has been proposed to function as a means for symbiotic bacteria to avoid digestion, or as a mechanism for the sponge to distinguish between food and symbionts (Thomas et al., 2010). The fact that the relatively phylogenetically distant SM17 and SM18 isolates possess orthologs of the same TPR, while their closest terrestrial relatives do not; suggests that this protein may indeed play a role in the symbiotic interactions between these bacteria and their sponge host *Haliclona simulans*.

#### ENA Gene Pool Genes Commonly Present in Other Environmental *Streptomyces* Isolates

In a similar fashion to the aforementioned analysis of orthologous genes, an additional analysis was performed, including the genomes from the other isolates previously determined to belong to the SM17 and SM18 phylogenetic clades (Figure 2). The aim was to assess whether genes present in the SM17 and SM18’s ENA gene pool are also present in other closely related relatives derived from other diverse environments, given the possibility that they may possess adaptations to their particular environmental niches that overlap with those identified in our marine sponge-associated SM17 and SM18 strains.

In the previously identified SM17 clade (Figure 2), in addition to its closely related terrestrial type-strain J1074, the clade also included the environmental isolates *Streptomyces albidoflavus* SM254, which was isolated from copper-rich subsurface fluids within an iron mine (Badalamenti et al., 2016); *Streptomyces sampsonii* KJ40, which was isolated from rhizosphere soil in a poplar plantation (Li et al., 2018); *Streptomyces koyangensis* VK-A60T, which was isolated from rhizosphere soil in a radish plantation (Lee et al., 2005); and *Streptomyces* sp. CLI2509, which is a fungus-derived isolate (Wyche et al., 2017). It is important to note that the SM17 clade also included the *Streptomyces* sp. FR-008 strain, however, this strain was not included in the analysis since it does not appear to be an environmental isolate, and it is a product of protoplast breeding of strains with little information in the literature regarding their isolation source (NCBI BioSample: SAMN03120580). The SM18 clade (Figure 2), in addition to its closely related terrestrial type-strain ATCC 33331, also included the environmental isolates *Streptomyces* sp. PAMC26508, which is an endosymbiotic bacterium isolated from the Antarctic lichen *Cladonia borealis* (Shin et al., 2013); *Streptomyces* sp. S501, isolated in sediment from a seaside wetland (NCBI BioSample: SAMN10144670); and *Streptomyces* sp. SirexAA-E, isolated from an insect/microbe symbiotic community (Bianchetti et al., 2013).

Interestingly, the majority of the genes present in the ENA gene pool were also present in the genomes of the other isolates. This is perhaps not surprising given the potential similarity in environmental stresses that these isolates may encounter, as the marine sponge-associated SM17 and SM18 strains; since they were all isolated from either (1) symbiotic communities, (2) high osmotic pressure environments and/or 3) aquatic environments. For example, the aforementioned *nuo* operon genes (Figure 7); potentially involved in adaptation to osmotic stress, are also present in the KJ40, the VK-A60T, and SM254 strains from the SM17 clade (Figure 2). It is well documented that osmoadaptation is an important trait possessed by rhizosphere-derived bacteria, since water uptake and exclusion of solutes such as Na^+^ and Cl^–^ by plants roots are likely to induce changes in osmolarity (Miller and Wood, 1996; Qurashi and Sabri, 2011), and for that reason salt-tolerant bacterium are commonly isolated from plant rhizospheres (Yuwono, 2005; Qurashi and Sabri, 2011). Thus, it is reasonable to assume that the presence of the *nuo* operon genes in the KJ40 and in the VK-A60T strains, both rhizosphere-derived isolates, may also be related to an increased resistance to osmotic stress, as it also seems to be the case to our marine sponge-derived isolates. Likewise, it is also possible that the SM254 strain, isolated from copper-rich subsurface fluids in an iron mine, will be exposed to osmotic stress and hence require appropriate adaptations to these conditions. Hence, it is plausible that the genes encoded in the *nuo* operon are not an adaptive response that is exclusively employed by some marine bacteria, as previously suggested (Penn and Jensen, 2012; Ian et al., 2014; Sun et al., 2018), but rather a more general mechanism of osmoadaptation that may be employed by bacteria in other environments as well.

Similarly, *proP* gene homologs were also present in all of the other genomes analyzed, with exception to the fungus-derived CLI2905 strain from the SM17 clade (Figure 2). Thus, given as has been previously discussed, that ProP acts both as an osmoregulator and as an osmosensor, together with transporting compatible solutes in *E. coli*; it may also be related to osmoadaptation in these isolates. These observations further highlight the potential adaptations which have been proposed in the ENA gene pool, that may be present in these other closely related relatives derived from other diverse environments, which may overlap with those identified in our marine sponge-associated SM17 and SM18 strains.

## Conclusion

The *Streptomyces* genus is exceptionally important when it comes to the identification and production of bioactive molecules, but those derived from the marine environment are currently particularly not well characterized. This study provides novel insights into possible ENAs employed by *Streptomyces* spp. isolated from marine sponges, and how these are potentially linked to diverse secondary metabolite biosynthesis. By providing high quality genomic information for the SM17 and SM18 strains isolated from *Haliclona simulans*, which have been previously shown to have antimicrobial activity against important pathogens, we were able to perform several comparative analyses with their terrestrial counterparts *S. albus* J1074 and *S. pratensis* ATCC 33331. The genomic analyses identified a diversity of putative smBGCs, which could potentially explain the previously determined antimicrobial activities reported for these marine isolates, such as smBGCs potentially encoding the production of candicidin, antimycin, SGR PTMs, surugamides, and mannopeptimycin, in SM17; and smBGCs potentially encoding the production of bafilomycin, SGR PTMs, curamycin, and caboxamycin, in SM18. Several smBGCs appear to be unique in the marine isolates in comparison to their terrestrial counterparts, which is particularly true in the case of the *Streptomyces* sp. SM18 isolate, when compared to *S. pratensis* ATCC 33331. Interestingly, while SM18 contains smBGCs encoding the production of siderophores such as coelichelin and mirubactin, it lacks the smBGC encoding the production of desferrioxamines; which is to our knowledge the first report of a *Streptomyces* isolate lacking this capacity. Comparative genomics analysis allowed us to identify genes that could be involved in mechanisms that may be relevant for their adaptation to their particular environmental niche, including resistance to osmotic stress; transcriptional regulation; symbiotic interactions; antimicrobial compounds production and resistance; ABC transporters; and HGT and other potential defense-related features. Expanding on the genetic knowledge of these organisms and their underlying mechanisms of adaptability is important, in not only allowing us to gain a better understanding of marine bacteria and their evolution, but also in helping with the discovery of potential new bioactive small molecules and in how to potentially manipulate and optimize their production.

## Data Availability

The complete genome sequences of SM17, SM18 and the plasmid sequences pSM17A, pSM17B, and pSM17C have been deposited in GenBank under the accession numbers CP029338, CP029342, CP029339, CP029340, and CP029341 respectively. The closest reference genomes used in this study for comparative purposes were *S. albus* J1074 (accession no. CP004370.1) and *S. pratensis* ATCC 33331 (accession no. CP002475.1).

## Author Contributions

EA, AC, SJ, and AD conceived and designed the experiments and analyzed the data. EA and AC performed the experiments. EA and AD wrote the manuscript.

## Conflict of Interest Statement

The authors declare that the research was conducted in the absence of any commercial or financial relationships that could be construed as a potential conflict of interest.

## Footnotes

1 https://sourceforge.net/projects/bbmap/

2 https://inkscape.org/
