# Corrigendum: Comparative Genomics of Marine Sponge-Derived *Streptomyces* spp. Isolates SM17 and SM18 With Their Closest Terrestrial Relatives Provides Novel Insights Into Environmental Niche Adaptations and Secondary Metabolite Biosynthesis Potential

**DOI:** 10.3389/fmicb.2019.02213

**Published:** 2019-09-24

**Authors:** Eduardo L. Almeida, Andrés Felipe Carrillo Rincón, Stephen A. Jackson, Alan D. W. Dobson

**Affiliations:** ^1^School of Microbiology, University College Cork, Cork, Ireland; ^2^Environmental Research Institute, University College Cork, Cork, Ireland

**Keywords:** comparative genomics, *Streptomyces*, environmental adaptation, marine sponge bacteria, secondary metabolite biosynthetic gene clusters, single molecule real-time sequencing

An author name was incorrectly spelled as “Rincón AFC”. The correct spelling is “Carrillo Rincón AF”.

The authors apologize for this error and state that this does not change the scientific conclusions of the article in any way. The original article has been updated.

