# Supplementary material for: Comparative Genomics of Marine Sponge-Derived Streptomyces spp. Isolates SM17 and SM18 With Their Closest Terrestrial Relatives Provides Novel Insights Into Environmental Niche Adaptations and Secondary Metabolite Biosynthesis Potential
Source: Front Microbiol. 2019 Jul 26;10:1713. doi: 10.3389/fmicb.2019.01713 (PMC6676996; doi:10.3389/fmicb.2019.01713)
Supplement: Supplementary file 1 [file Table_1.DOCX]

**Table S1:** Putative smBGCs predicted to be present in the SM17 genome using the antiSMASH program.

| **Cluster** | **Type** | **From** | **To** | **Most similar known cluster** |
| --- | --- | --- | --- | --- |
| Cluster 1 | Lantipeptide-T1pks-Nrps | 9520 | 282674 | Candicidin biosynthetic gene cluster (100% of genes show similarity) |
| Cluster 2 | T3pks | 287634 | 328731 | Herboxidiene biosynthetic gene cluster (12% of genes show similarity) |
| Cluster 3 | Bacteriocin-Terpene | 454168 | 486617 | Carotenoid biosynthetic gene cluster (54% of genes show similarity) |
| Cluster 4 | Ectoine | 1226907 | 1237305 | Ectoine biosynthetic gene cluster (100% of genes show similarity) |
| Cluster 5 | Siderophore | 2136943 | 2148763 | Desferrioxamine B biosynthetic gene cluster (100% of genes show similarity) |
| Cluster 6 | Nrps | 2377319 | 2421668 | - |
| Cluster 7 | Nrps | 2881828 | 2987506 | Mannopeptimycin biosynthetic gene cluster (7% of genes show similarity) |
| Cluster 8 | Nrps | 3238171 | 3288460 | Scabichelin biosynthetic gene cluster (40% of genes show similarity) |
| Cluster 9 | Nrps | 4012785 | 4073245 | Mannopeptimycin biosynthetic gene cluster (51% of genes show similarity) |
| Cluster 10 | Lantipeptide | 4096267 | 4118852 | SAL-2242_biosynthetic_gene_cluster (100% of genes show similarity) |
| Cluster 11 | Thiopeptide | 4422066 | 4454538 | - |
| Cluster 12 | Terpene | 4948699 | 4969673 | Albaflavenone biosynthetic gene cluster (100% of genes show similarity) |
| Cluster 13 | Terpene | 5280779 | 5303064 | Kanamycin biosynthetic gene cluster (1% of genes show similarity) |
| Cluster 14 | Siderophore | 5558173 | 5573204 | - |
| Cluster 15 | Nrps | 5648771 | 5708652 | Tetronasin biosynthetic gene cluster (9% of genes show similarity) |
| Cluster 16 | Bacteriocin | 5946086 | 5957414 | - |
| Cluster 17 | Bacteriocin | 6364321 | 6374536 | - |
| Cluster 18 | Terpene | 6440077 | 6466641 | Hopene biosynthetic gene cluster (76% of genes show similarity) |
| Cluster 19 | T1pks-Nrps | 6501121 | 6550531 | SGR PTMs biosynthetic gene cluster (100% of genes show similarity) |
| Cluster 20 | Terpene-Nrps | 6690082 | 6777548 | Lividomycin biosynthetic gene cluster (10% of genes show similarity) |
