# Supplementary material for: Comparative Genomics of Marine Sponge-Derived Streptomyces spp. Isolates SM17 and SM18 With Their Closest Terrestrial Relatives Provides Novel Insights Into Environmental Niche Adaptations and Secondary Metabolite Biosynthesis Potential
Source: Front Microbiol. 2019 Jul 26;10:1713. doi: 10.3389/fmicb.2019.01713 (PMC6676996; doi:10.3389/fmicb.2019.01713)
Supplement: Supplementary file 2 [file Table_2.DOCX]

**Table S2:** Putative smBGCs predicted to be present in the SM18 genome using the antiSMASH program.

| **Cluster** | **Type** | **From** | **To** | **Most similar known cluster** |
| --- | --- | --- | --- | --- |
| Cluster 1 | Terpene | 29172 | 50203 | - |
| Cluster 2 | T2pks-Terpene | 68208 | 117510 | Spore pigment biosynthetic gene cluster (75% of genes show similarity) |
| Cluster 3 | Melanin | 151502 | 161990 | Melanin biosynthetic gene cluster (100% of genes show similarity) |
| Cluster 4 | T1pks | 390670 | 438115 | Surfactin biosynthetic gene cluster (8% of genes show similarity) |
| Cluster 5 | Nrps | 457604 | 520442 | Daptomycin biosynthetic gene cluster (9% of genes show similarity) |
| Cluster 6 | Lantipeptide | 596609 | 619990 | - |
| Cluster 7 | T1pks-Nrps | 728498 | 820279 | Chivosazole biosynthetic gene cluster (22% of genes show similarity) |
| Cluster 8 | Terpene | 902952 | 924010 | Steffimycin biosynthetic gene cluster (19% of genes show similarity) |
| Cluster 9 | Ectoine | 1363227 | 1373625 | Ectoine biosynthetic gene cluster (100% of genes show similarity) |
| Cluster 10 | T1pks-Otherks | 2295950 | 2349406 | - |
| Cluster 11 | Lantipeptide-Lassopeptide | 2379602 | 2423090 | - |
| Cluster 12 | Butyrolactone | 3482738 | 3493514 | Griseoviridin/viridogrisein biosynthetic gene cluster (8% of genes show similarity) |
| Cluster 13 | T1pks | 4955466 | 5003964 | Arginomycin biosynthetic gene cluster (20% of genes show similarity) |
| Cluster 14 | Bacteriocin | 5043642 | 5055585 | - |
| Cluster 15 | Terpene | 5125104 | 5146111 | Carbapenem MM 4550 biosynthetic gene cluster (10% of genes show similarity) |
| Cluster 16 | Siderophore | 5488861 | 5503466 | - |
| Cluster 17 | Otherks-Nrps | 5660928 | 5786416 | A33853 biosynthetic gene cluster (43% of genes show similarity) |
| Cluster 18 | Thiopeptide-Terpene | 5857201 | 5913709 | Isorenieratene biosynthetic gene cluster (100% of genes show similarity) |
| Cluster 19 | T2pks | 5974917 | 6017450 | Pristinamycin biosynthetic gene cluster (21% of genes show similarity) |
| Cluster 20 | Bacteriocin | 6097616 | 6109004 | - |
| Cluster 21 | Nrps | 6308570 | 6362642 | Coelibactin biosynthetic gene cluster (100% of genes show similarity) |
| Cluster 22 | Terpene | 6757753 | 6784324 | Hopene biosynthetic gene cluster (69% of genes show similarity) |
| Cluster 23 | Nrps | 6978302 | 7029194 | Coelichelin biosynthetic gene cluster (100% of genes show similarity) |
| Cluster 24 | T1pks-Nrps | 7030460 | 7111803 | Herboxidiene biosynthetic gene cluster (16% of genes show similarity) |
| Cluster 25 | T1pks | 7248399 | 7346791 | Bafilomycin biosynthetic gene cluster (100% of genes show similarity) |
| Cluster 26 | Nrps | 7527142 | 7577107 | Mirubactin biosynthetic gene cluster (50% of genes show similarity) |
