# Supplementary material for: Comparative Genomics of Marine Sponge-Derived Streptomyces spp. Isolates SM17 and SM18 With Their Closest Terrestrial Relatives Provides Novel Insights Into Environmental Niche Adaptations and Secondary Metabolite Biosynthesis Potential
Source: Front Microbiol. 2019 Jul 26;10:1713. doi: 10.3389/fmicb.2019.01713 (PMC6676996; doi:10.3389/fmicb.2019.01713)
Supplement: Supplementary file 3 [file Table_3.DOCX]

**Table S3:** Putative smBGCs predicted to be present in the *S. albus* J1074 genome using the antiSMASH program**.**

| **Cluster** | **Type** | **From** | **To** | **Most similar known cluster** |
| --- | --- | --- | --- | --- |
| Cluster 1 | T1pks-Nrps | 3011 | 61711 | - |
| Cluster 2 | T1pks-Nrps | 224752 | 274162 | SGR PTMs biosynthetic gene cluster (100% of genes show similarity) |
| Cluster 3 | Terpene | 308626 | 335190 | Hopene biosynthetic gene cluster (76% of genes show similarity) |
| Cluster 4 | Bacteriocin | 415688 | 425903 | - |
| Cluster 5 | Otherks | 669953 | 711002 | Avermectin biosynthetic gene cluster (66% of genes show similarity) |
| Cluster 6 | Bacteriocin | 879961 | 891289 | - |
| Cluster 7 | Nrps | 1136316 | 1199422 | Tetronasin biosynthetic gene cluster (9% of genes show similarity) |
| Cluster 8 | Siderophore | 1268164 | 1283196 | - |
| Cluster 9 | Terpene | 1531774 | 1554059 | Kanamycin biosynthetic gene cluster (1% of genes show similarity) |
| Cluster 10 | Terpene | 1865241 | 1886215 | Albaflavenone biosynthetic gene cluster (100% of genes show similarity) |
| Cluster 11 | Thiopeptide | 2376688 | 2409159 | - |
| Cluster 12 | Bacteriocin | 2560714 | 2571226 | Goadsporin biosynthetic gene cluster (12% of genes show similarity) |
| Cluster 13 | Lantipeptide | 2694944 | 2735867 | SAL-2242 biosynthetic gene cluster (100% of genes show similarity) |
| Cluster 14 | Nrps | 3553726 | 3604015 | Scabichelin biosynthetic gene cluster (40% of genes show similarity) |
| Cluster 15 | Nrps | 3877105 | 3982777 | Mannopeptimycin biosynthetic gene cluster (7% of genes show similarity) |
| Cluster 16 | Nrps | 4469477 | 4513826 | - |
| Cluster 17 | Siderophore | 4740450 | 4752270 | Desferrioxamine B biosynthetic gene cluster (100% of genes show similarity) |
| Cluster 18 | Ectoine | 5635346 | 5645744 | Ectoine biosynthetic gene cluster (100% of genes show similarity) |
| Cluster 19 | Other | 6337383 | 6381213 | Indigoidine biosynthetic gene cluster (80% of genes show similarity) |
| Cluster 20 | Bacteriocin-Terpene | 6396161 | 6430146 | Carotenoid biosynthetic gene cluster (54% of genes show similarity) |
| Cluster 21 | T3pks | 6520374 | 6561471 | Herboxidiene biosynthetic gene cluster (12% of genes show similarity) |
| Cluster 22 | Lantipeptide-T1pks-Nrps | 6566423 | 6838639 | Candicidin biosynthetic gene cluster (100% of genes show similarity) |
