# Supplementary material for: Comparative Genomics of Marine Sponge-Derived Streptomyces spp. Isolates SM17 and SM18 With Their Closest Terrestrial Relatives Provides Novel Insights Into Environmental Niche Adaptations and Secondary Metabolite Biosynthesis Potential
Source: Front Microbiol. 2019 Jul 26;10:1713. doi: 10.3389/fmicb.2019.01713 (PMC6676996; doi:10.3389/fmicb.2019.01713)
Supplement: Supplementary file 4 [file Table_4.DOCX]

**Table S4:** Putative smBGCs predicted to be present in the *S. pratensis* ATCC 33331 genome using the antiSMASH program**.**

| **Cluster** | **Type** | **From** | **To** | **Most similar known cluster** |
| --- | --- | --- | --- | --- |
| Cluster 1 | Blactam-T1pks-Nrps | 70873 | 221343 | Carbapenem MM 4550 biosynthetic gene cluster (65% of genes show similarity) |
| Cluster 2 | Nrps | 327693 | 378596 | Coelichelin biosynthetic gene cluster (90% of genes show similarity) |
| Cluster 3 | Terpene | 393138 | 418630 | Isorenieratene biosynthetic gene cluster (100% of genes show similarity) |
| Cluster 4 | Bacteriocin | 557521 | 568045 | - |
| Cluster 5 | Blactam | 634715 | 658209 | Clavulanic acid biosynthetic gene cluster (20% of genes show similarity) |
| Cluster 6 | Terpene | 738599 | 765184 | Hopene biosynthetic gene cluster (69% of genes show similarity) |
| Cluster 7 | T1pks | 1104244 | 1198984 | Vicenistatin biosynthetic gene cluster (60% of genes show similarity) |
| Cluster 8 | Bacteriocin | 1409256 | 1420548 | - |
| Cluster 9 | Nrps | 1560425 | 1622443 | Arylomycin biosynthetic gene cluster (22% of genes show similarity) |
| Cluster 10 | Siderophore | 1894420 | 1909004 | - |
| Cluster 11 | Terpene | 1973589 | 1994659 | - |
| Cluster 12 | Bacteriocin | 2151745 | 2161966 | - |
| Cluster 13 | Butyrolactone | 3819031 | 3829957 | Lactonamycin biosynthetic gene cluster (3% of genes show similarity) |
| Cluster 14 | T1pks-Nrps | 4023172 | 4080102 | Istamycin biosynthetic gene cluster (11% of genes show similarity) |
| Cluster 15 | Siderophore | 4727646 | 4739427 | Desferrioxamine B biosynthetic gene cluster (83% of genes show similarity) |
| Cluster 16 | Lantipeptide | 4792335 | 4815403 | - |
| Cluster 17 | Terpene | 5226078 | 5247109 | - |
| Cluster 18 | Ectoine | 5705549 | 5715947 | Ectoine biosynthetic gene cluster (100% of genes show similarity) |
| Cluster 19 | T2pks-Otherks | 6079056 | 6136319 | Cinerubin B biosynthetic gene cluster (28% of genes show similarity) |
| Cluster 20 | Terpene | 6181067 | 6202113 | Steffimycin biosynthetic gene cluster (19% of genes show similarity) |
| Cluster 21 | Ectoine-Terpene | 6482662 | 6509101 | Ectoine biosynthetic gene cluster (100% of genes show similarity) |
| Cluster 22 | Bacteriocin | 6532212 | 6542439 | - |
| Cluster 23 | T3pks | 6695745 | 6736803 | Tetronasin biosynthetic gene cluster (11% of genes show similarity) |
| Cluster 24 | Melanin | 6830784 | 6841248 | Melanin biosynthetic gene cluster (100% of genes show similarity) |
| Cluster 25 | T2pks-Terpene | 6882032 | 6931236 | Spore pigment biosynthetic gene cluster (83% of genes show similarity) |
| Cluster 26 | Nrps | 7082613 | 7132319 | Zorbamycin biosynthetic gene cluster (6% of genes show similarity) |
| Cluster 27 | Butyrolactone | 7211688 | 7222623 | - |
