# Supplementary material for: Comparative Genomics of Marine Sponge-Derived Streptomyces spp. Isolates SM17 and SM18 With Their Closest Terrestrial Relatives Provides Novel Insights Into Environmental Niche Adaptations and Secondary Metabolite Biosynthesis Potential
Source: Front Microbiol. 2019 Jul 26;10:1713. doi: 10.3389/fmicb.2019.01713 (PMC6676996; doi:10.3389/fmicb.2019.01713)
Supplement: Supplementary file 5 [file Table_5.DOCX]

**Table S5:** List of orthologous genes and their respective annotations (excluding hypothetical proteins), which are commonly present in the sponge-derived isolates SM17 and SM18, while absent in their terrestrial counterparts J1074 and ATCC 33331. When the gene name was not determined, a generic unique name was given (group_XXXX) by the Roary program. ‘*’ - genes without multiple copies or paralogs in the terrestrial isolates’ genomes, considering only the ones with a defined gene name; ‘^a^’ - genes for which both sponge-derived isolates presented a higher copy number in comparison to their terrestrial counterparts; ‘^b^’ -genes for which the SM17 isolate had a higher copy number in comparison to its terrestrial counterpart *S. albus* J1074; ‘^c^’ - genes for which the SM18 isolate had a higher copy number in comparison to its terrestrial counterpart *S. pratensis* ATCC 33331.

| **Gene** | **Annotations** |
| --- | --- |
| *adhD*^a^ | NDMA-dependent alcohol dehydrogenase / Zinc-binding alcohol dehydrogenase |
| *ahcY*^a^ | Adenosylhomocysteinase |
| *aprX** | Serine protease AprX / Subtilase family protein / Peptidase S8 |
| *bepR** | HTH-type transcriptional repressor BepR / TetR family transcriptional regulator |
| *bioC*^b^ | Malonyl-[acyl-carrier protein] O-methyltransferase / Class I SAM-dependent methyltransferase |
| *cpnA** | Cyclopentanol dehydrogenase / SDR family oxidoreductase |
| *cynR*^a^ | HTH-type transcriptional regulator CynR / LysR family transcriptional regulator |
| *degU*^a^ | Transcriptional regulatory protein DegU / DNA-binding response regulator |
| *fccA** | Fumarate reductase flavoprotein subunit / FAD-dependent oxidoreductase |
| *folQ** | Putative DHNTP pyrophosphohydrolase / NUDIX hydrolase |
| group_1044 | Integrase core domain / IS3 family transposase |
| group_1217 | Fumarylacetoacetase |
| group_1272 | Toxin-antitoxin system, RelE family |
| group_1944 | Restriction endonuclease |
| group_1945 | IS3 family transposase |
| group_5008 | Alpha-ketoglutaric semialdehyde dehydrogenase / NADP-dependent aldehyde dehydrogenase |
| group_5198 | Aminoglycoside phosphotransferase |
| group_5212 | NADH:flavin oxidoreductase |
| group_5385 | Aminoglycoside phosphotransferase |
| group_5540 | DUF3307 domain-containing protein |
| group_5542 | Phosphohydrolase |
| group_5643 | ATP/GTP-binding protein |
| group_5772 | Tetratricopeptide repeat protein |
| group_5776 | Radical SAM protein |
| group_5789 | Darcynin |
| group_5793 | Amine oxidase, flavin-containing |
| group_5796 | Transcriptional regulator, IclR family |
| group_5798 | Nuclear transport factor 2 family protein |
| group_5803 | Nuclear transport factor 2 family protein |
| group_5818 | Acyltransferase 3 |
| group_5819 | Transcriptional regulator PadR-like family protein |
| group_5821 | ABC transporter permease |
| group_5836 | Acyltransferase |
| *hmgA*^a^ | Homogentisate 1,2-dioxygenase |
| *hsdA** | 3-alpha-hydroxysteroid dehydrogenase/carbonyl reductase / SDR family oxidoreductase |
| *htpG*^c^ | Chaperone protein HtpG / heat shock protein 90 |
| *liaS*^a^ | HPK7 family sensor histidine kinase LiaS |
| *mftC*^b^ | Putative mycofactocin radical SAM maturase MftC / radical SAM protein |
| *ndx1** | NUDIX hydrolase |
| *nuoA*^a^ | NADH-quinone oxidoreductase subunit A |
| *nuoH*^a^ | NADH-quinone oxidoreductase subunit H |
| *nuoJ*^a^ | NADH-quinone oxidoreductase subunit J |
| *nuoK*^a^ | NADH-quinone oxidoreductase subunit K |
| *nuoL*^a^ | NADH-quinone oxidoreductase subunit L |
| *nuoM*^a^ | NADH-quinone oxidoreductase subunit M |
| *nuoN*^a^ | NADH-quinone oxidoreductase subunit N |
| *proP*^a^ | Proline/betaine transporter |
| *ptsG* | PTS system glucose-specific EIICB component |
| *rhmR*^a^ | HTH-type transcriptional regulator KipR / MarR family transcriptional regulator |
| *scoA** | 3-oxoacid CoA-transferase, A subunit |
| *scoB*^a^ | 3-oxoacid CoA-transferase, B subunit |
| *ssuE*^b^ | FMN reductase (NADPH) |
| *tauB*^a^ | Aliphatic sulfonates import ATP-binding protein SsuB / ABC transporter ATP-binding protein |
| *tcrA*^a^ | Transcriptional regulatory protein CutR / DNA-binding response regulator |
| *xecD*^a^ | SDR family oxidoreductase |
| *yihX** | Alpha-D-glucose 1-phosphate phosphatase YihX / HAD family phosphatase |
| *yknY*^a^ | Uncharacterised ABC transporter ATP-binding protein YknY |
