# Supplementary material for: Comparative Genomics of Marine Sponge-Derived Streptomyces spp. Isolates SM17 and SM18 With Their Closest Terrestrial Relatives Provides Novel Insights Into Environmental Niche Adaptations and Secondary Metabolite Biosynthesis Potential
Source: Front Microbiol. 2019 Jul 26;10:1713. doi: 10.3389/fmicb.2019.01713 (PMC6676996; doi:10.3389/fmicb.2019.01713)
Supplement: Supplementary file 6 [file Table_6.DOCX]

**Table S6:** Nucleotide sequence identity comparison between the partial *nuo*-operon present in the marine isolates *Streptomyces* sp. SM17, *Streptomyces* sp. SM18, *Salinispora arenicola* CNS-205, *Salinispora tropica* CNB-440, and *Kocuria flava* S43, obtained using discontiguous MegaBLAST alignments.

|  | *Streptomyces* sp. SM17 | *Streptomyces* sp. SM18 | *Salinispora arenicola* CNS-205 | *Salinispora tropica* CNB-440 | *Kocuria flava* S43 |
| --- | --- | --- | --- | --- | --- |
| *Streptomyces* sp. SM17 | - | 70% | 69% | 69% | 70% |
| *Streptomyces* sp. SM18 | 70% | - | 68% | 69% | 68% |
| *Salinispora arenicola* CNS-205 | 69% | 68% | - | 86% | 73% |
| *Salinispora tropica* CNB-440 | 69% | 69% | 86% | - | 73% |
| *Kocuria flava* S43 | 70% | 68% | 73% | 73% | - |
