# Supplementary figures and images for: Comparative Genomics of Marine Sponge-Derived Streptomyces spp. Isolates SM17 and SM18 With Their Closest Terrestrial Relatives Provides Novel Insights Into Environmental Niche Adaptations and Secondary Metabolite Biosynthesis Potential
Source: Front Microbiol. 2019 Jul 26;10:1713. doi: 10.3389/fmicb.2019.01713 (PMC6676996; doi:10.3389/fmicb.2019.01713)

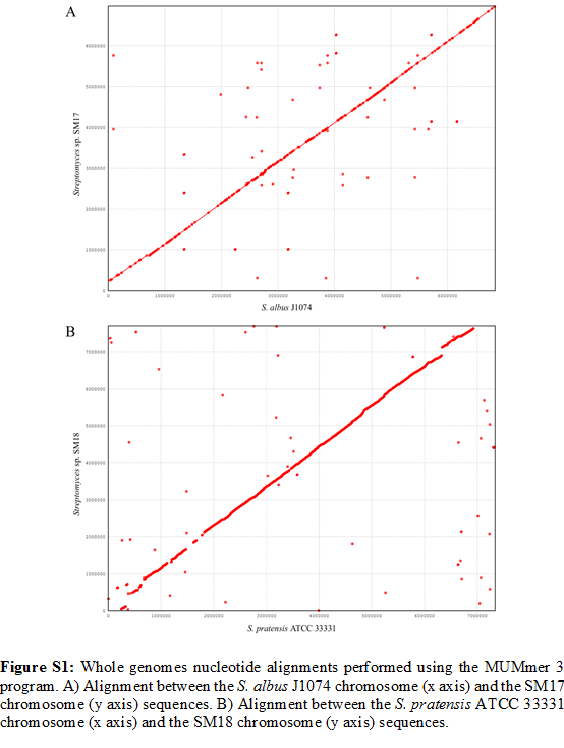

Supplement: Supplementary file 7 [file Image_1.tif]

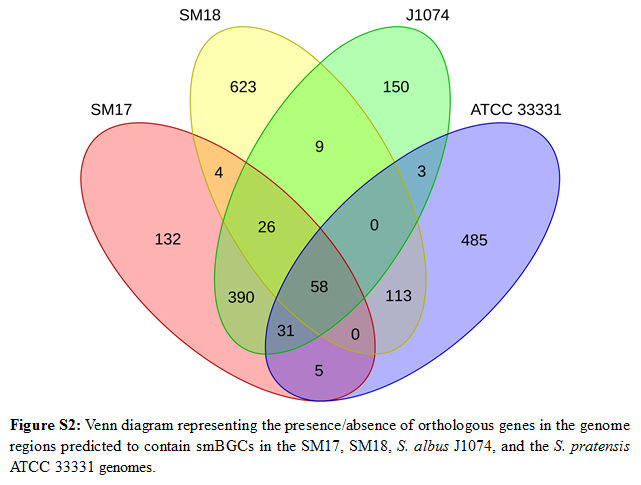

Supplement: Supplementary file 8 [file Image_2.tif]
